# Supplementary material for: Antimicrobial Diterpene Alkaloids from an Agelas citrina Sponge Collected in the Yucatán Peninsula
Source: Mar Drugs. 2022 Apr 28;20(5):298. doi: 10.3390/md20050298 (PMC9143306; doi:10.3390/md20050298)
Supplement: Supplementary file 1 [file marinedrugs-20-00298-s001.zip › marinedrugs-1701671-supplementary.pdf]

SUPPLEMENTARY MATERIALS

Antimicrobial Diterpene Alkaloids from an *Agelas citrina* Sponge Collected in Yucatán Peninsula

Dawrin Pech-Puch[1,2], Abel M. Forero[1], Juan Carlos Fuentes-Monteverde[3], Cristina Lasarte-Monterrubio[4], Marta Martinez-Guitian[4], Carlos González-Salas[2], Sergio Guillén-Hernández[2], Harold Villegas-Hernández[2], Alejandro Beceiro[4], Christian Griesinger[3], Jaime Rodríguez[1,*] and Carlos Jiménez [1,*]

[1] Departamento de Química, Facultade de Ciencias e Centro de Investigacións Científicas Avanzadas (CICA), Universidade de A Coruña, 15071 A Coruña, Spain
[2] Departamento de Biología Marina, Universidad Autónoma de Yucatán, Km. 15.5, carretera Mérida-Xmatkuil, A.P. 4-116 Itzimná, Merida C.P. 97100, Yucatán,Mexico
[3] Department of NMR Based Structural Biology, Max Planck Institute (MPI) for Multidisciplinary Sciences, Am Fassberg 11, 37077 Göttingen, Germany
[4] Microbiology Department of the University Hospital A Coruña (CHUAC), Institute of Biomedical Re-search of A Coruña (INIBIC), Centro de Investigación Biomédica en Red (CIBER) Infec., 15006 A Coruña, Spain

*Correspondence: jaime.rodriguez@udc.es; carlos.jimenez@udc.es

Scheme S1. Extraction and fractionation scheme. 4

Figure S1. ^{1}H NMR spectrum (500 MHz, DMSO-d_6) of 8-epiagelasine T (**1**). 5

Figure S2.^{13}C NMR spectrum (125 MHz, DMSO-d_6) of 8-epiagelasine T (**1**). 5

Figure S3. HSQC spectrum (500 MHz, DMSO-d_6) of 8-epiagelasine T (**1**). 6

Figure S4. ^{1}H-^{1}H COSY spectrum (500 MHz, DMSO-d_6) of 8-epiagelasine T (**1**). 6

Figure S5. HMBC spectrum (500 MHz, DMSO-d_6) of 8-epiagelasine T (**1**). 7

Figure S6. 1D-NOESY spectrum irradiating on CH_3-17 (δ_H 1.00) of 8-epiagelasine T (**1**) (500 MHz, DMSO-d_6). 7

Figure S7. 1D-NOESY spectrum irradiating on CH_3-8 (δ_H 0.84) and CH_3-20 (δ_H 0.74) of 8-epiagelasine T (**1**) (500 MHz, DMSO-d_6). 8

Figure S8. NOESY spectrum of 8-epiagelasine T (**1**) (500 MHz, DMSO-d_6). 8

Figure S9. (+)-HR-ESIMS of 8-epiagelasine T (**1**). 9

Table S1. ^{1}H NMR (500 MHz) and ^{13}C NMR (125 MHz) and ^{1}H NMR (800 MHz) and ^{13}C NMR (200 MHz)* spectral data for 10-epiagelasine B (**2**) in $CDCl_3$ and C_6D_6. 10

Figure S10. ^{1}H NMR spectrum (500 MHz, $CDCl_3$) of 10-epiagelasine B (**2**). 11

Figure S11.^{13}C NMR spectrum (125 MHz, $CDCl_3$) of 10-epiagelasine B (**2**). 11

Figure S12. HSQC spectrum (500 MHz, $CDCl_3$) of 10-epiagelasine B (**2**). 12

Figure S13. ^{1}H-^{1}H COSY spectrum (500 MHz, $CDCl_3$) of 10-epiagelasine B (**2**). 12

Figure S14. HMBC spectrum (500 MHz, $CDCl_3$) of 10-epiagelasine B (**2**). 13

Figure S15. NOESY spectrum (500 MHz, $CDCl_3$) of 10-epiagelasine B (**2**). 13

Figure S16. (+)-HR-ESIMS of 10-epiagelasine B (**2**). 14

Figure S17. ^{1}H NMR spectrum (500 MHz, C_6D_6) of 10-epiagelasine B (**2**). 14

Figure S18. ^{13}C NMR spectrum (125 MHz, C_6D_6) of 10-epiagelasine B (**2**). 15

Figure S19. HSQC NMR spectrum (500 MHz, C_6D_6) of 10-epiagelasine B (**2**). 15

Figure S20. ^{1}H-^{1}H COSY NMR spectrum (500 MHz, C_6D_6) of 10-epiagelasine B (**2**). 16

Figure S21. HMBC NMR spectrum (500 MHz, C_6D_6) of 10-epiagelasine B (**2**). 16

Figure S22. NOESY NMR spectrum (500 MHz, C_6D_6) of 10-epiagelasine B (**2**). 17

Figure S23. ^{1}H NMR spectrum (800 MHz, $CDCl_3$, after 24 h) of 10-epiagelasine B (**2**). 17

Figure S24.^{13}C NMR spectrum (200 MHz, $CDCl_3$, after 24 h) of 10-epiagelasine B (**2**). 18

Figure S25. HSQC spectrum (800 MHz, $CDCl_3$, after 24 h) of 10-epiagelasine B (**2**). NUS parameters: 20%/384/38. 18

Figure S26. ^{1}H-^{1}H COSY spectrum (800 MHz, $CDCl_3$, after 24 h) of 10-epiagelasine B (**2**). NUS parameters: 50%/384/192. 19

Figure S27. HMBC spectrum (800 MHz, $CDCl_3$, after 24 h) of 10-epiagelasine B (**2**). NUS parameters: 25%/512/64. 19

Figure S28. 1D-NOESY spectrum irradiating on CH_3-17 (δ_H 1.10) of 10-epiagelasine B (**2**) (800 MHz, $CDCl_3$, after 24 h). 20

Figure S29. 1D-NOESY spectrum irradiating on CH_3-19 (δ_H 1.15) of 10-epiagelasine B (**2**) (800 MHz, $CDCl_3$, after 24 h). 20

Figure S30. 1D-NOESY spectrum irradiating on CH_3-20 (δ_H 0.87) of 10-epiagelasine B (**2**) (800 MHz, $CDCl_3$, after 24 h). 21

Figure S31. 2D IPAP-HSQMBC spectrum (optimized to 6 Hz) after selective inversion of H-10 proton of 10-epi-agelasine B (A). Slice from C-19 to H-10 (B). Measurement of $^{2,3}J_{CH}$ values were performed by analysis of IPAP multiplet patterns. NUS parameters: 12%/512/30. 22

Figure S32. 2D IPAP-HSQMBC spectrum (optimized to 6 Hz) after selective inversion of H-5 proton of **7**. Slice from C-7 to H-5 (B). Measurement of $^{2,3}J_{CH}$ values were performed by analysis of IPAP multiplet patterns. NUS parameters: 12%/512/30. 22

Figure S33. 2D IPAP-HSQMBC spectrum (optimized to 6 Hz) after selective inversion of H-5 proton of **8**. Slice from C-7 to H-5 (B). Measurement of $^{2,3}J_{CH}$ values were performed by analysis of IPAP multiplet patterns. NUS parameters: 12%/512/30. 23

Figure S34. ^{1}H NMR spectrum (500 MHz, $CDCl_3$) of 12-hydroxyagelasidine C (**3**). 24

Figure S35. ^{13}C NMR spectrum (125 MHz, $CDCl_3$) of 12-hydroxyagelasidine C (**3**). 24

Figure S36. HSQC spectrum (500 MHz, $CDCl_3$) of 12-hydroxyagelasidine C (**3**). 25

Figure S37. ^{1}H-^{1}H COSY spectrum (500 MHz, $CDCl_3$) of 12-hydroxyagelasidine C (**3**). 25

Figure S38. HMBC spectrum (500 MHz, $CDCl_3$) of 12-hydroxyagelasidine C (**3**). 26

Figure S39. NOESY spectrum (500 MHz, $CDCl_3$) of 12-hydroxyagelasidine C (**3**). 26

Figure S40. (+)-HR-ESIMS of 12-hydroxyagelasidine C (**3**) 27

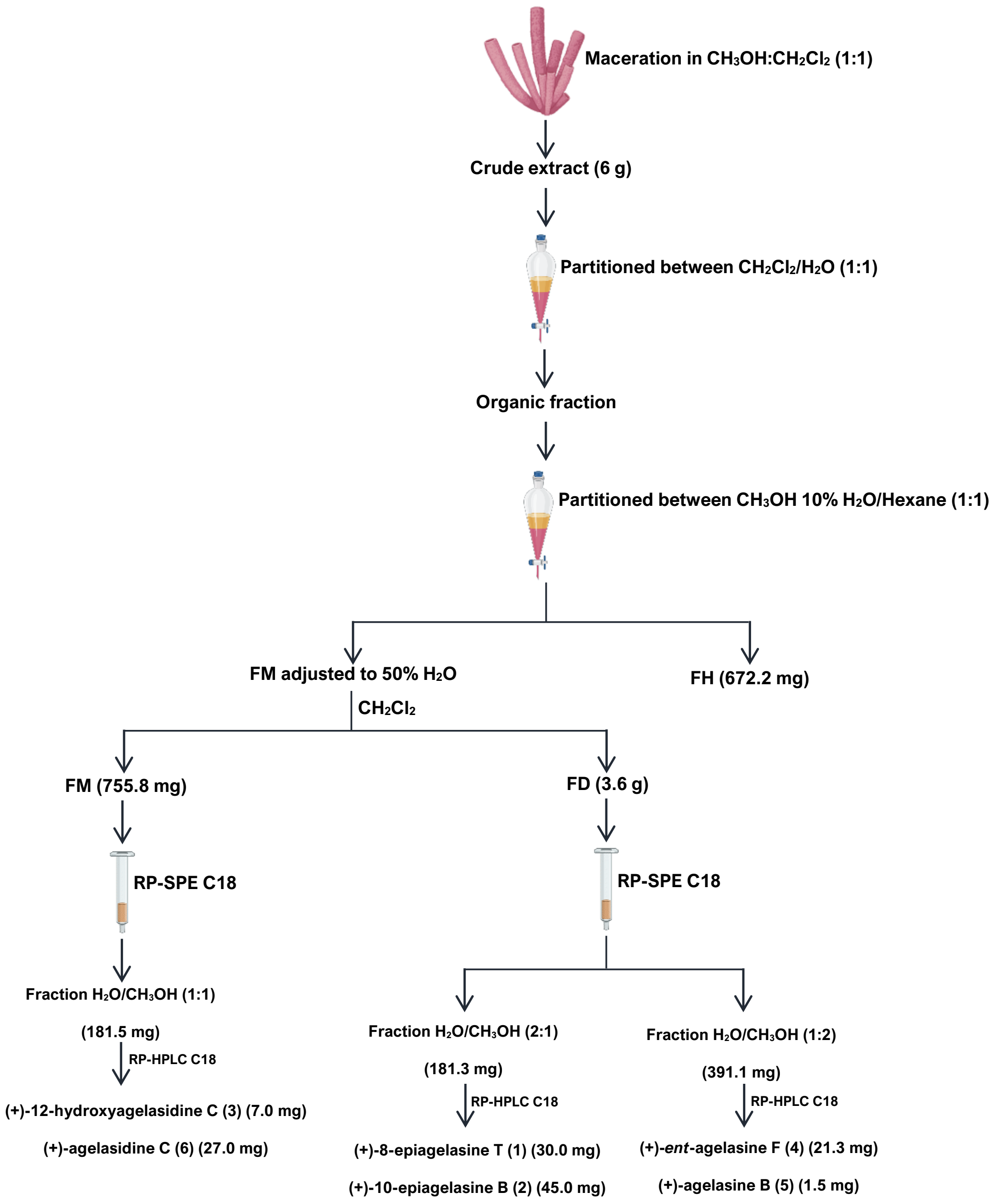

Scheme S1. Extraction and fractionation scheme.

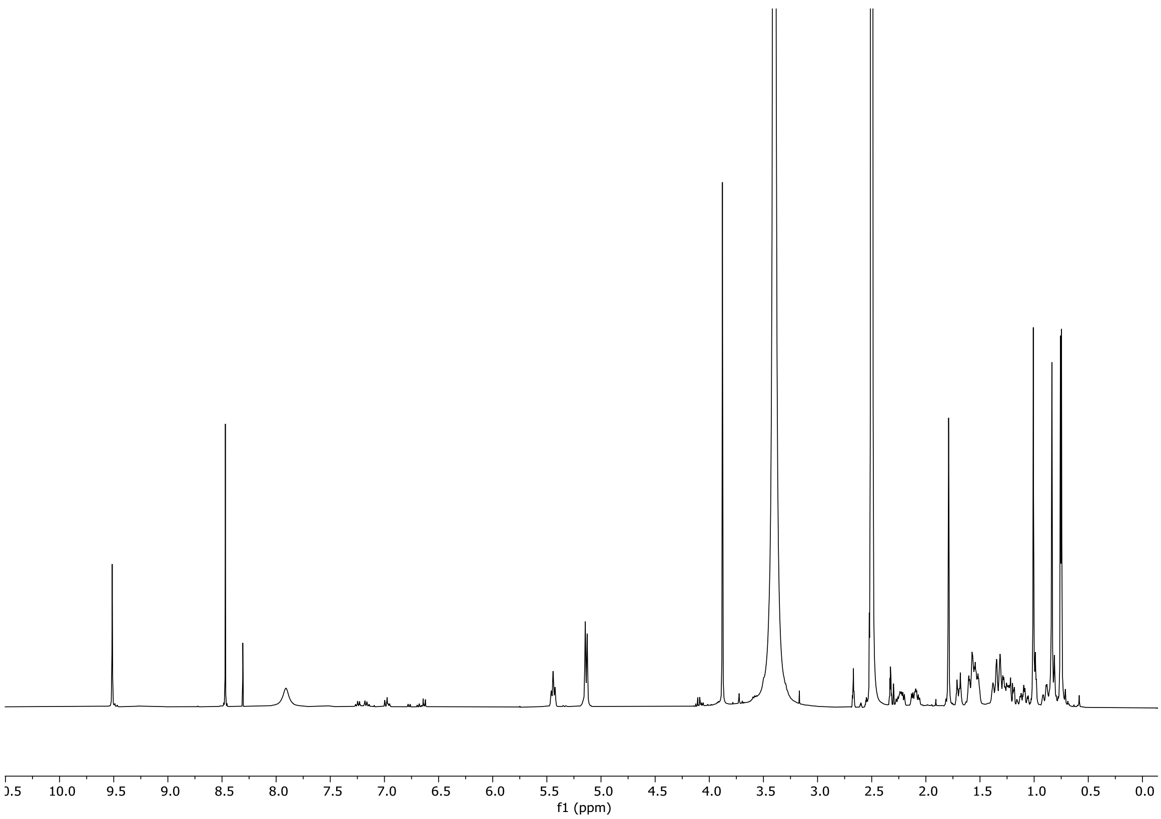

Figure S1. ^{1}H NMR spectrum (500 MHz, DMSO-d_6) of 8-epiagelasine T (**1**).

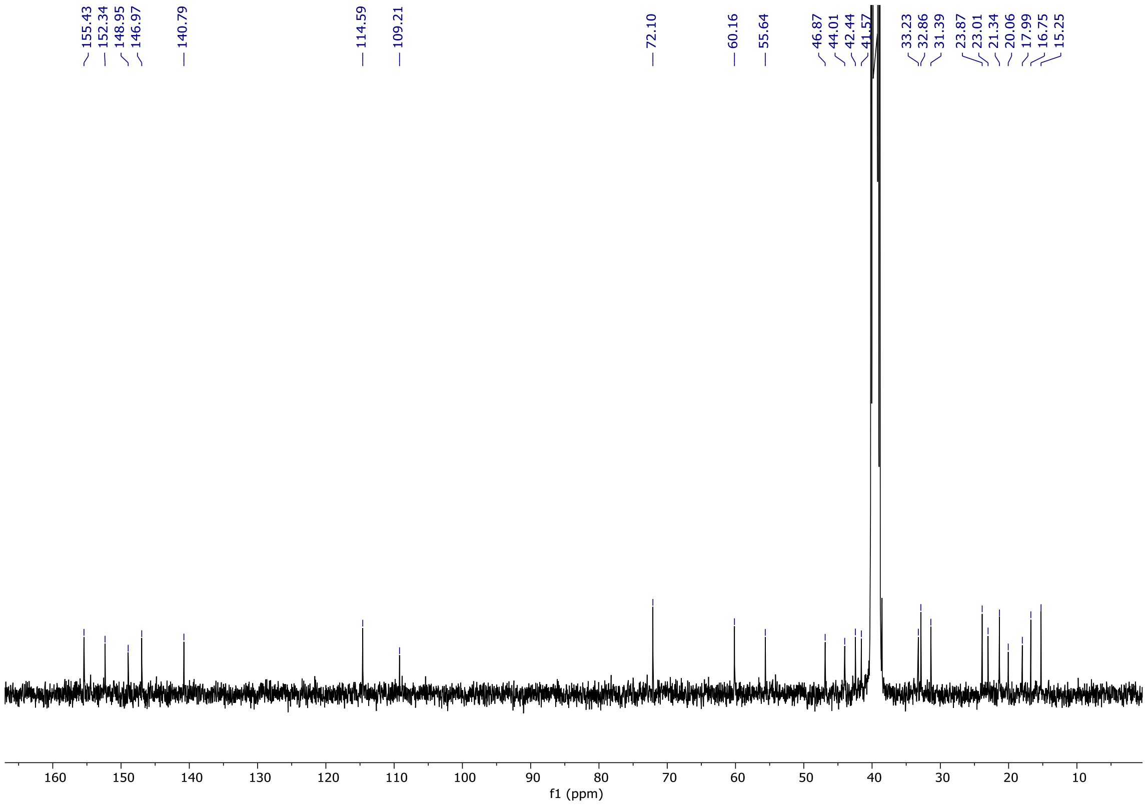

Figure S2. ^{13}C NMR spectrum (125 MHz, DMSO-d_6) of 8-epiagelasine T (**1**).

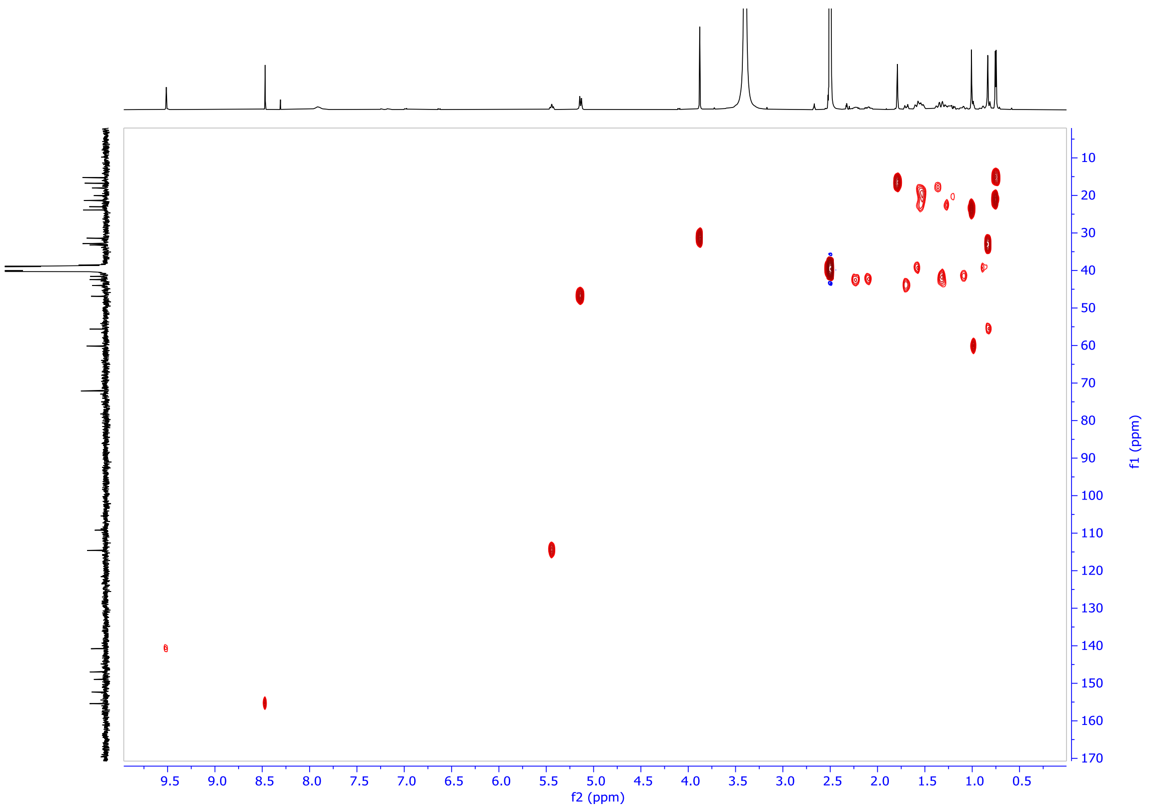

Figure S3. HSQC spectrum (500 MHz, DMSO-d_6) of 8-epiagelasine T (**1**).

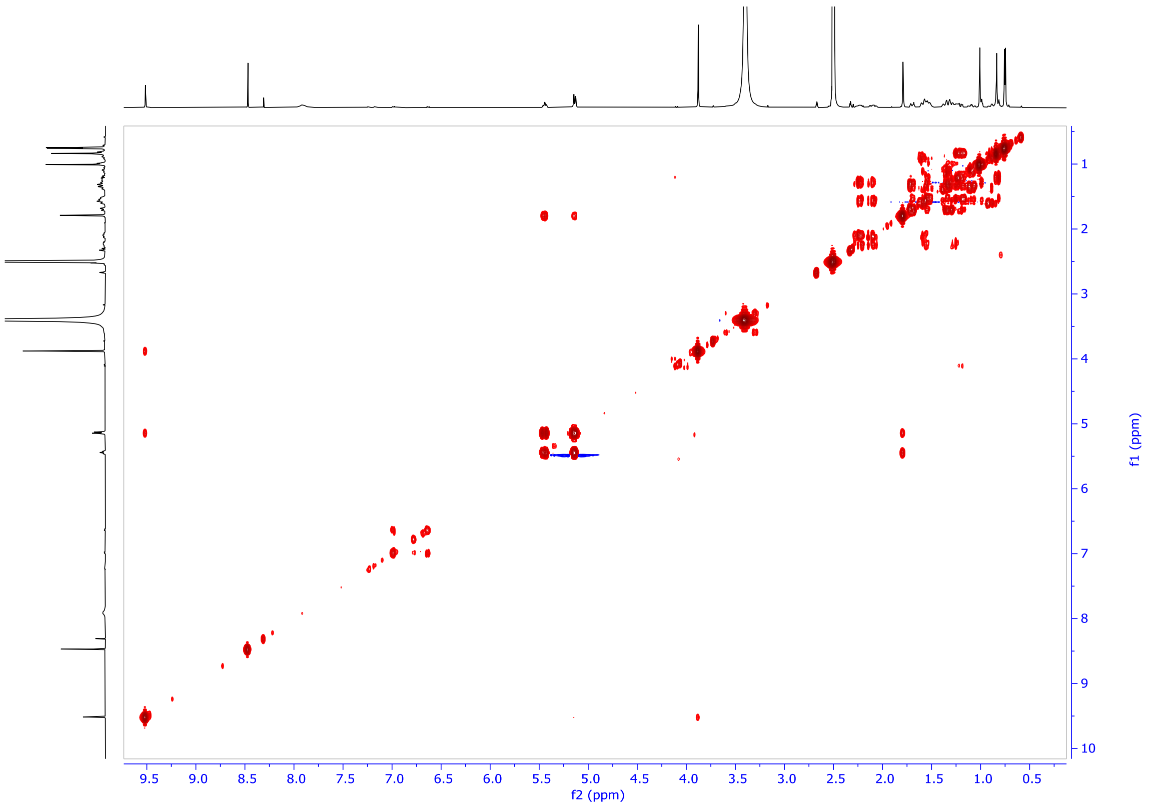

Figure S4. ^{1}H-^{1}H COSY spectrum (500 MHz, DMSO-d_6) of 8-epiagelasine T (**1**).

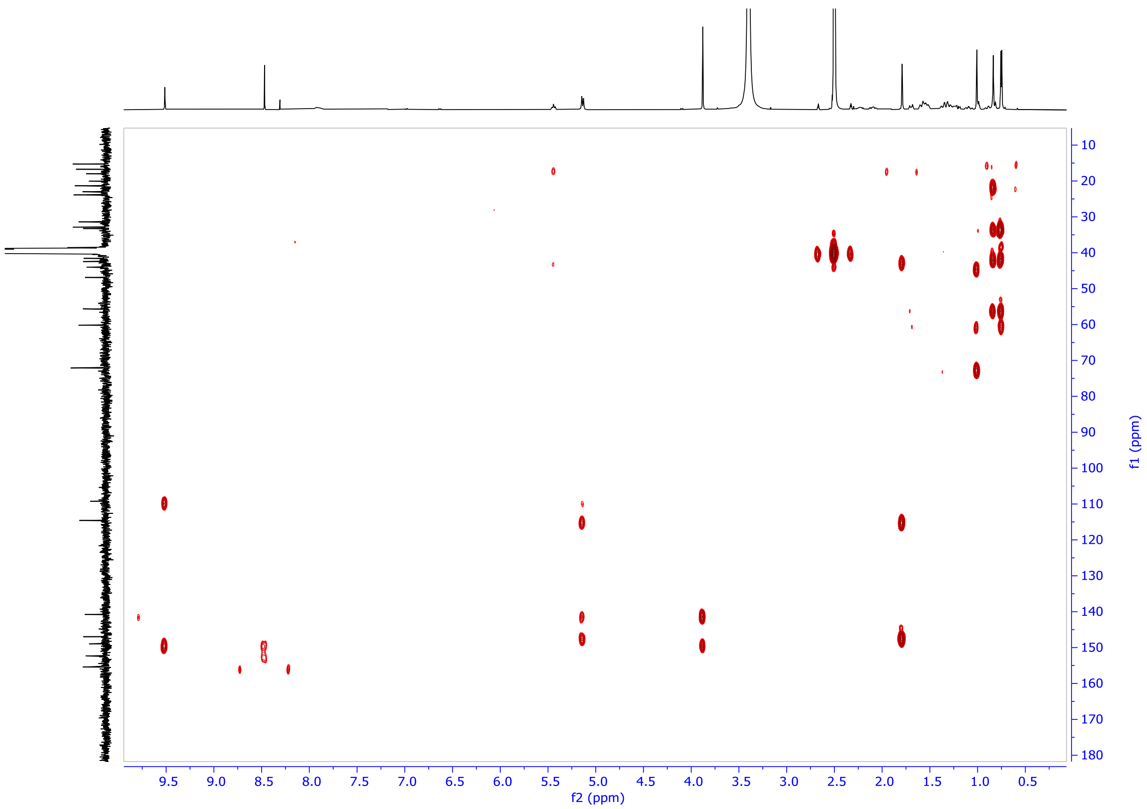

Figure S5. HMBC spectrum (500 MHz, DMSO-d_6) of 8-epiagelasine T (**1**).

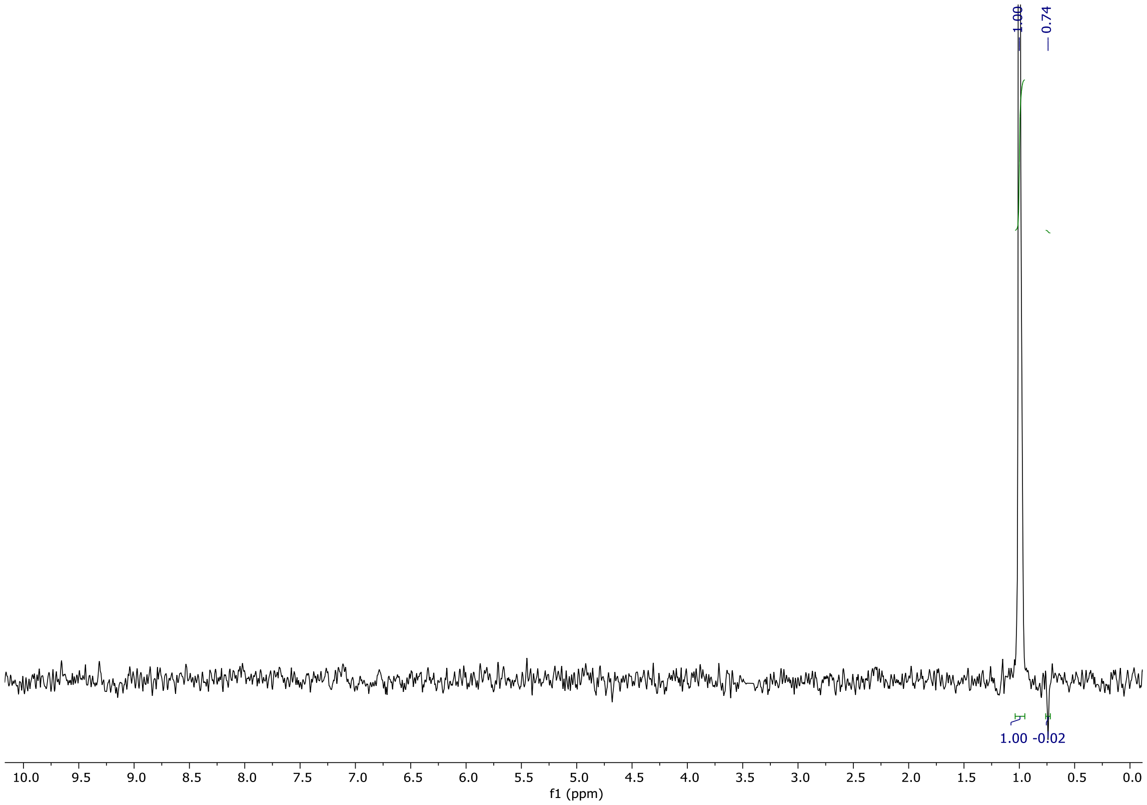

Figure S6. 1D-NOESY spectrum irradiating on CH_3-17 (δ_H 1.00) of 8-epiagelasine T (**1**) (500 MHz, DMSO-d_6).

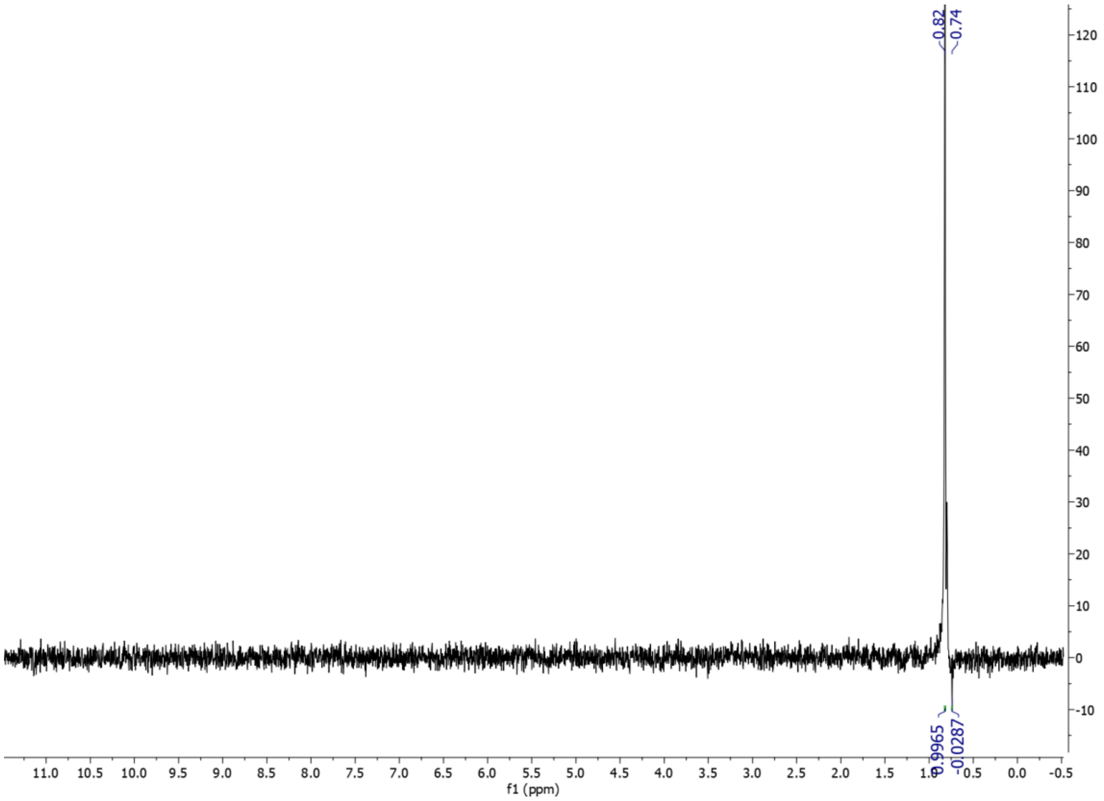

Figure S7. 1D-NOESY spectrum irradiating on CH_3-8 (δ_H 0.84) and CH_3-20 (δ_H 0.74) of 8-epiagelasine T (**1**) (500 MHz, DMSO-d_6).

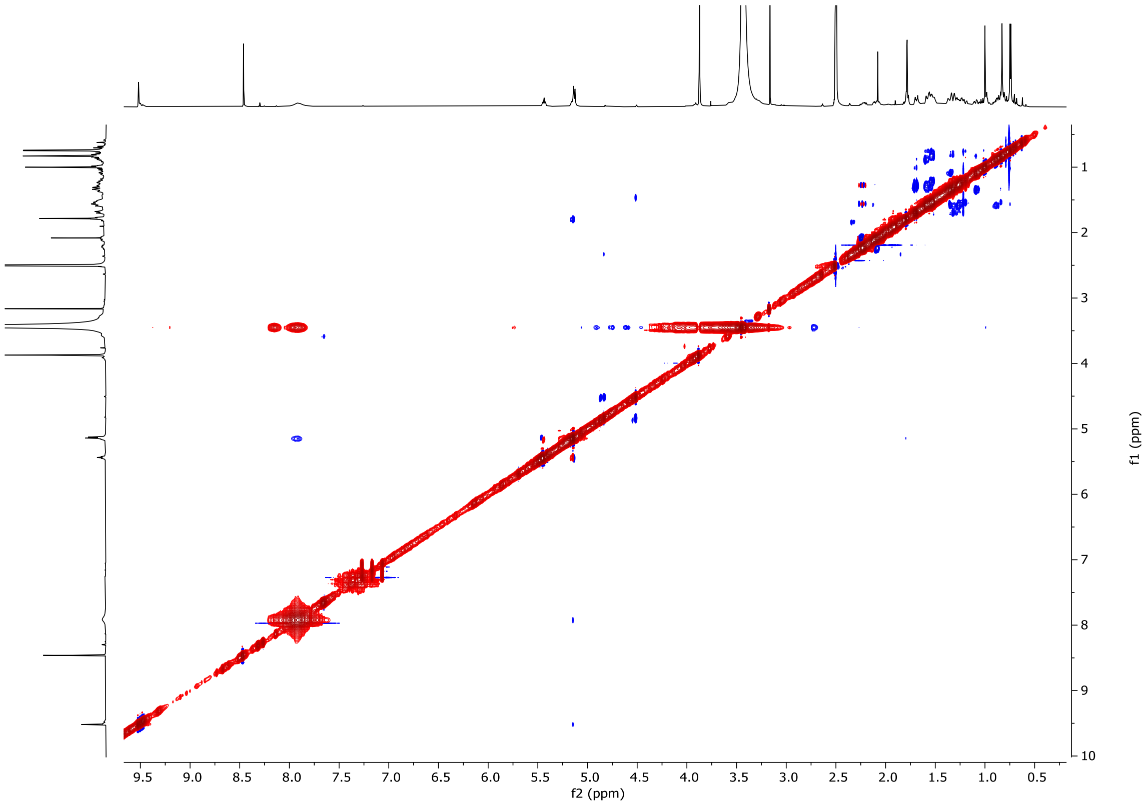

Figure S8. NOESY spectrum of 8-epiagelasine T (**1**) (500 MHz, DMSO-d_6).

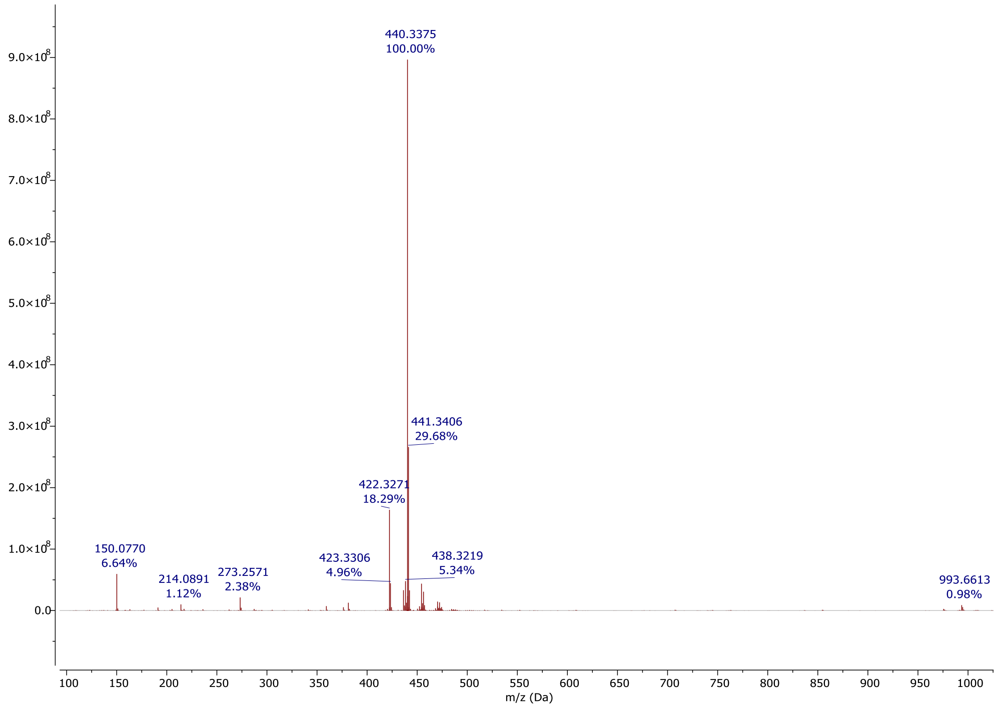

Figure S9. (+)-HR-ESIMS of 8-epiagelasine T (**1**).

Table S1. ^{1}H NMR (500 MHz) and ^{13}C NMR (125 MHz) and ^{1}H NMR (800 MHz) and ^{13}C NMR (200 MHz)* spectral data for 10-epiagelasine B (**2**) in $CDCl_3$ and C_6D_6.

Position	10-epiagelasine B[a]		10-epiagelasine B[b]		10-epiagelasine B[c]	
	δ_H, mult, (*J* in Hz)	δ_C, Type	δ_H, mult, (*J* in Hz)	δ_C, Type	δ_H, mult, (*J* in Hz)	δ_C, Type
1	1.64 m<br>1.33, m	19.7, CH_2	1.60, m<br>1.31, m	20.1, CH_2	1.65 m<br>1.24, m	31.0, CH_2
2	1.98, m<br>1.91, m	22.9, CH_2	1.98, m<br>1.21, m	23.4, CH_2	1.93, m<br>1.78, m	32.5, CH_2
3	5.30, brs	120.7, CH	5.37, brs	121.1, CH	5.20, brs	126.0, CH
4		136.2, C		136.5, C		134.0, C
5		40.5, C		39.4, C		42.6, C
6	1.80, m<br>1.71, m	33.7, CH_2	1.50, m<br>1.34, m	30.3, CH_2	2.39, dt (15.0, 4.1)<br>2.10, m	20.8, CH_2
7	1.64, m<br>1.42, m	29.2, CH_2	1.95, m<br>1.70, m	29.5, CH_2	1.60, m<br>1.31, m	28.4, CH_2
8	1.31, m	33.5, CH	1.40, m	33.7, CH	1.66, m	34.0, CH
9		39.1, C		40.8, C		41.0, C
10	2.28, brd, (12.9)	42.0, CH	2.38, d, (12.9)	42.3, CH	1.25, m	41.8, CH
11	1.35, m<br>1.25, m	30.0, CH_2	1.90, m<br>1.40, m	33.9, CH_2	1.58, m<br>1.38, m	35.5, CH_2
12	2.03, dt, (11.8, 5.6)<br>1.97, m	35.1, CH_2	2.01, m	35.4, CH_2	2.00, dd, (13.3, 7.8)<br>1.94, dd, (14.0, 7.0)	35.2, CH_2
13		149.2, C		149.1, C		152.2, C
14	5.45, t, (6.4)	115.0, CH	5.42, t, (6.8)	114.8, CH	5.50, m	112.7, CH
15	5.25, d, (6.5)	48.6, CH_2	5.11, d, (6.6)	48.2, CH_2	5.52, brs	50.2, CH_2
16	1.86, s	17.2, CH_3	1.80, s	16.7, CH_3	1.84, s	17.1, CH_3
17	1.07, d, (7.4)	18.7, CH_3	1.10, d, (7.4)	18.9, CH_3	1.10, d, (7.3)	17.5, CH_3
18	1.62, s	21.7, CH_3	1.67, s	22.0, CH_3	1.62, s	19.9, CH_3
19	0.86, s	17.2, CH_3	1.01, s	15.9, CH_3	1.15, s	24.3, CH_3
20	0.94, s	15.6, CH_3	0.90, s	18.6, CH_3	0.87, s	18.3, CH_3
2′	8.54, s	155.8, CH	8.31, s	155.5, CH	8.71, s	149.0, CH
4′		149.7, C		149.4, C		143.5, C
5′		110.0, C		110.0, C		119.7, C
6′		151.8, C		152.2, C		148.7, C
8′	9.87, s	142.2, C	9.30, s	141.0, CH	9.90, s	143.3, C
9′-*N*-Me	4.06, s	31.9, CH_3	3.30, s	31.0, CH_3	4.11, s	34.0, CH_3

[a]In $CDCl_3$. [b]In C_6D_6. [c]In $CDCl_3$ (after 24 h)

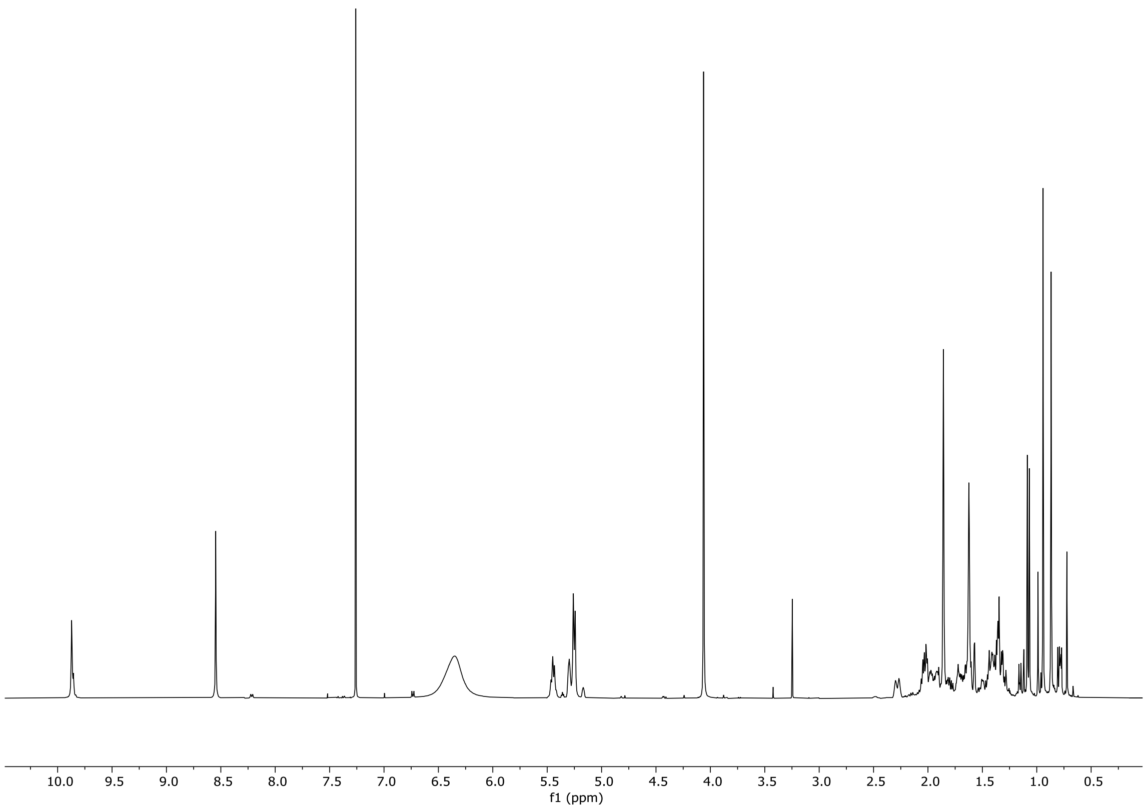

Figure S10. ^{1}H NMR spectrum (500 MHz, $CDCl_3$) of 10-epiagelasine B (**2**).

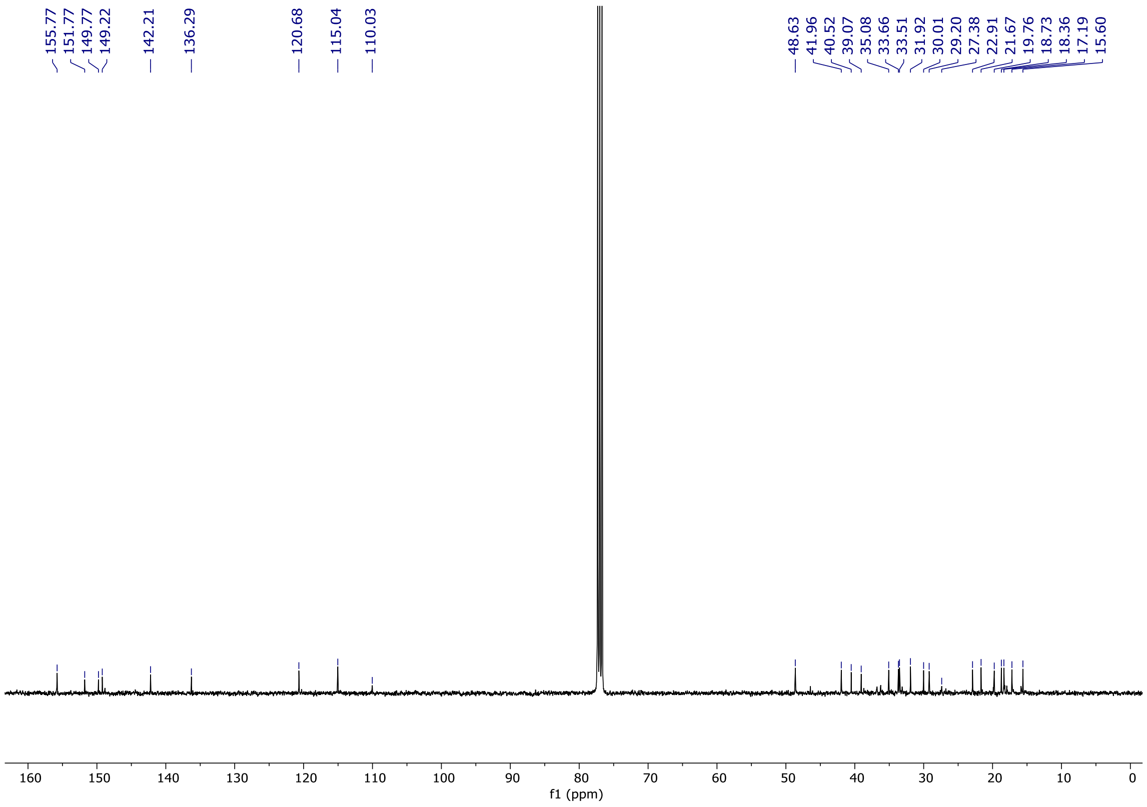

Figure S11. ^{13}C NMR spectrum (125 MHz, $CDCl_3$) of 10-epiagelasine B (**2**).

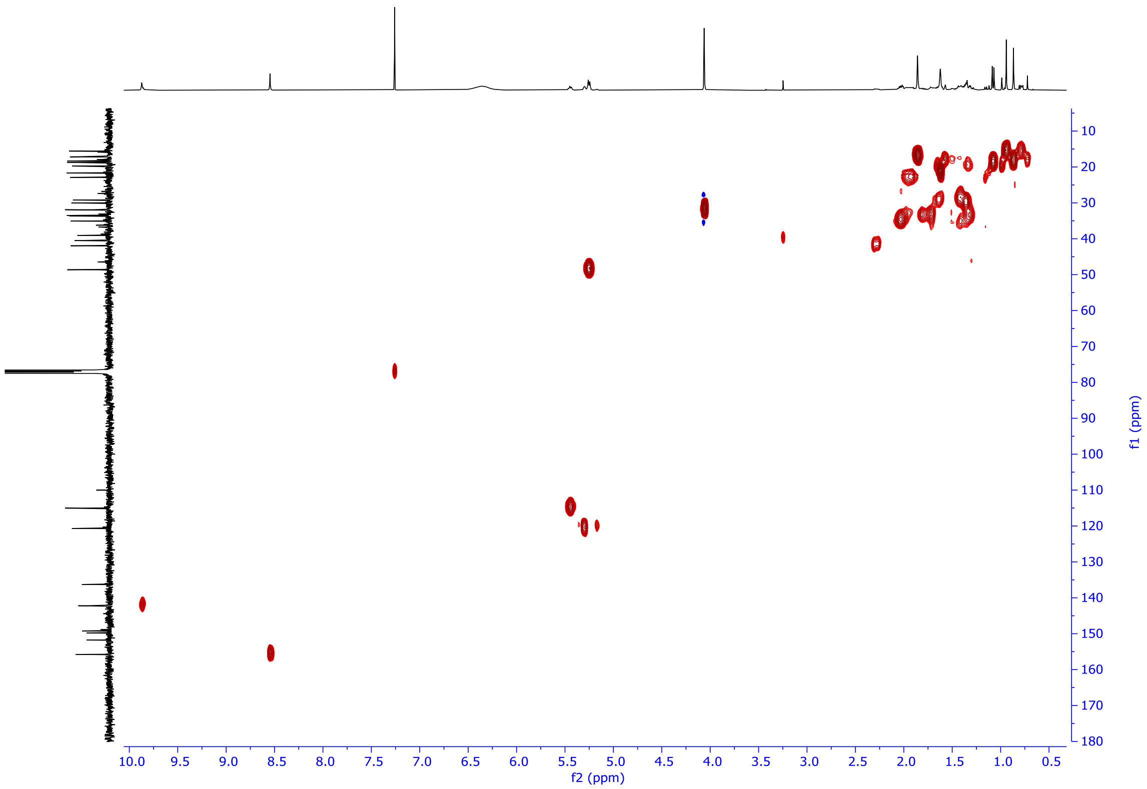

Figure S12. HSQC spectrum (500 MHz, $CDCl_3$) of 10-epiagelasine B (**2**).

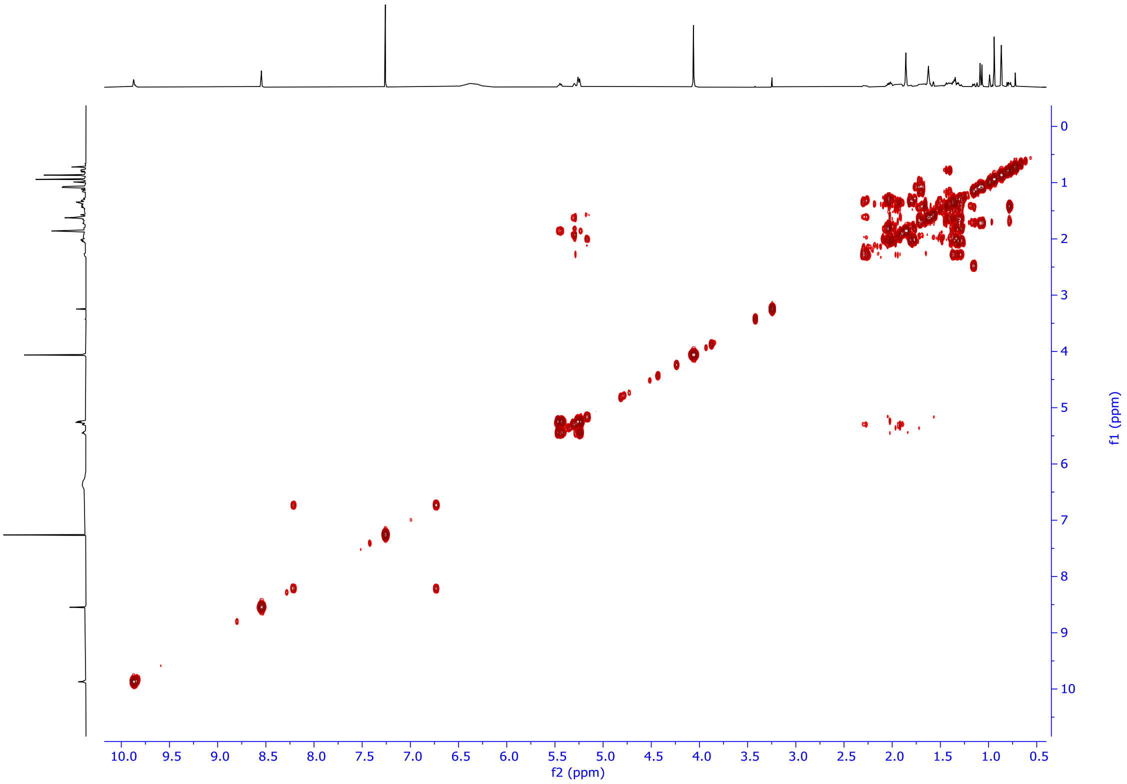

Figure S13. ^1H-^1H COSY spectrum (500 MHz, $CDCl_3$) of 10-epiagelasine B (**2**).

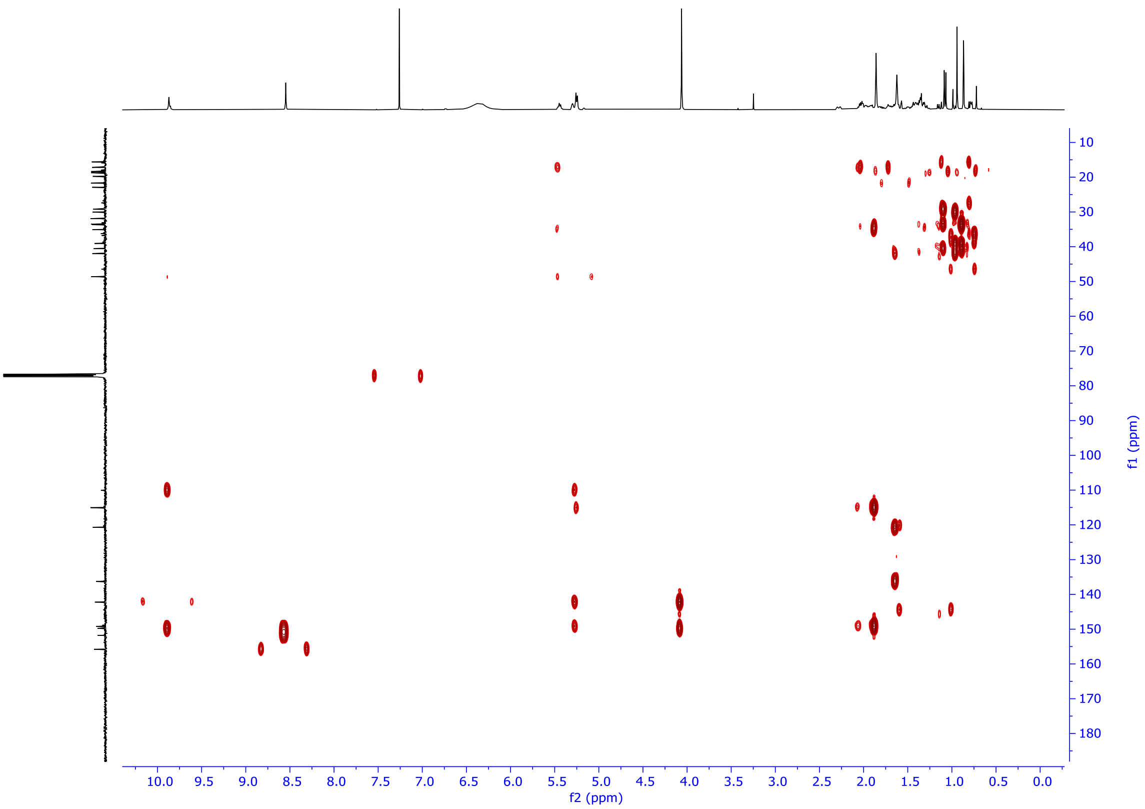

Figure S14. HMBC spectrum (500 MHz, $CDCl_3$) of 10-epiagelasine B (**2**).

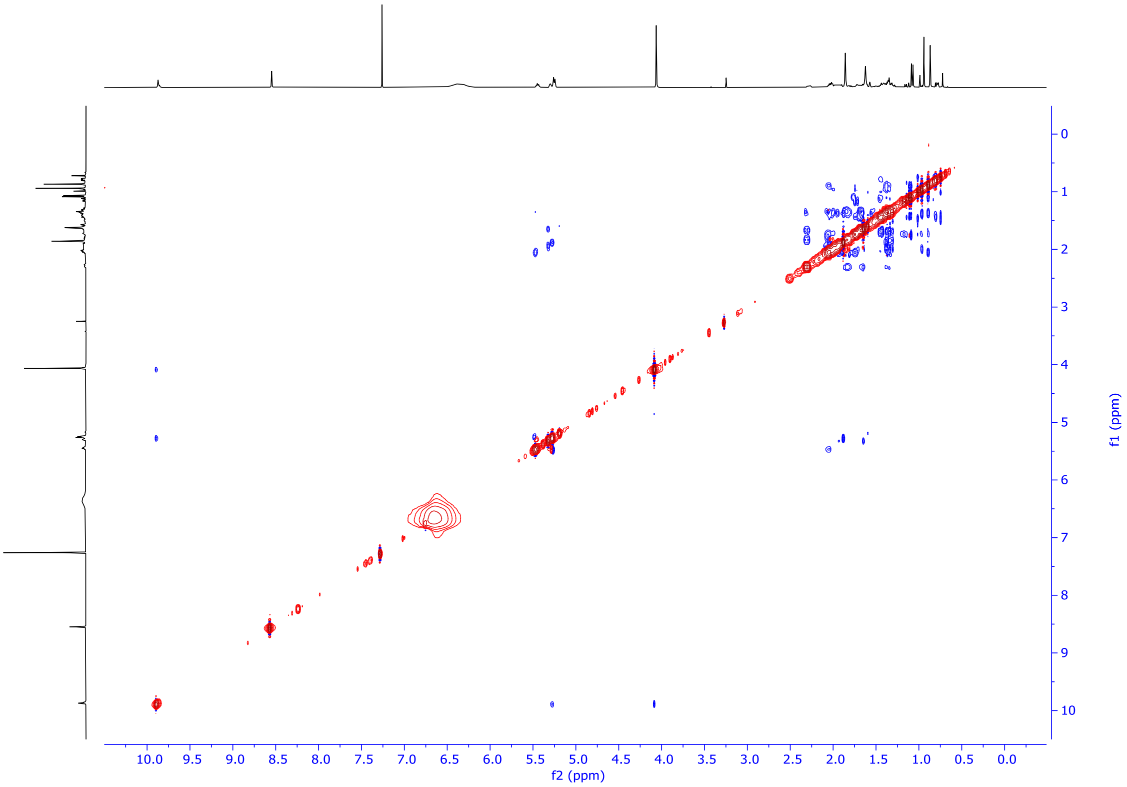

Figure S15. NOESY spectrum (500 MHz, $CDCl_3$) of 10-epiagelasine B (**2**).

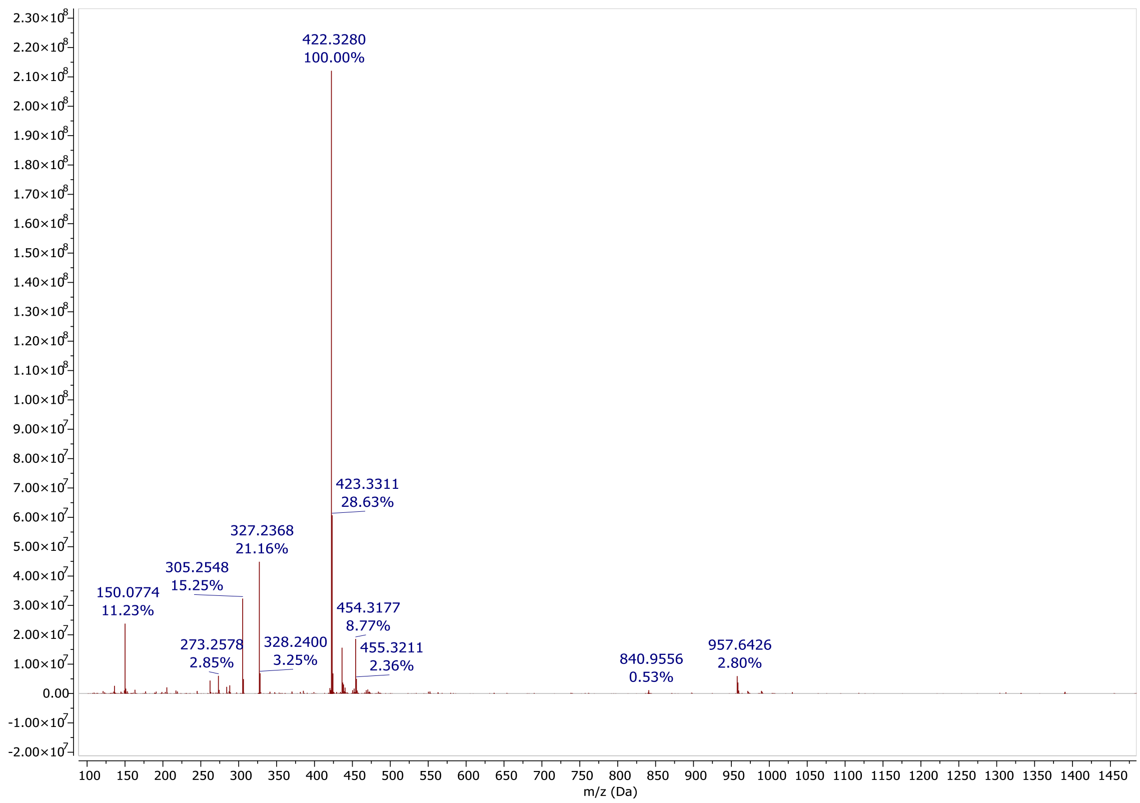

Figure S16. (+)-HR-ESIMS of 10-epiagelasine B (**2**).

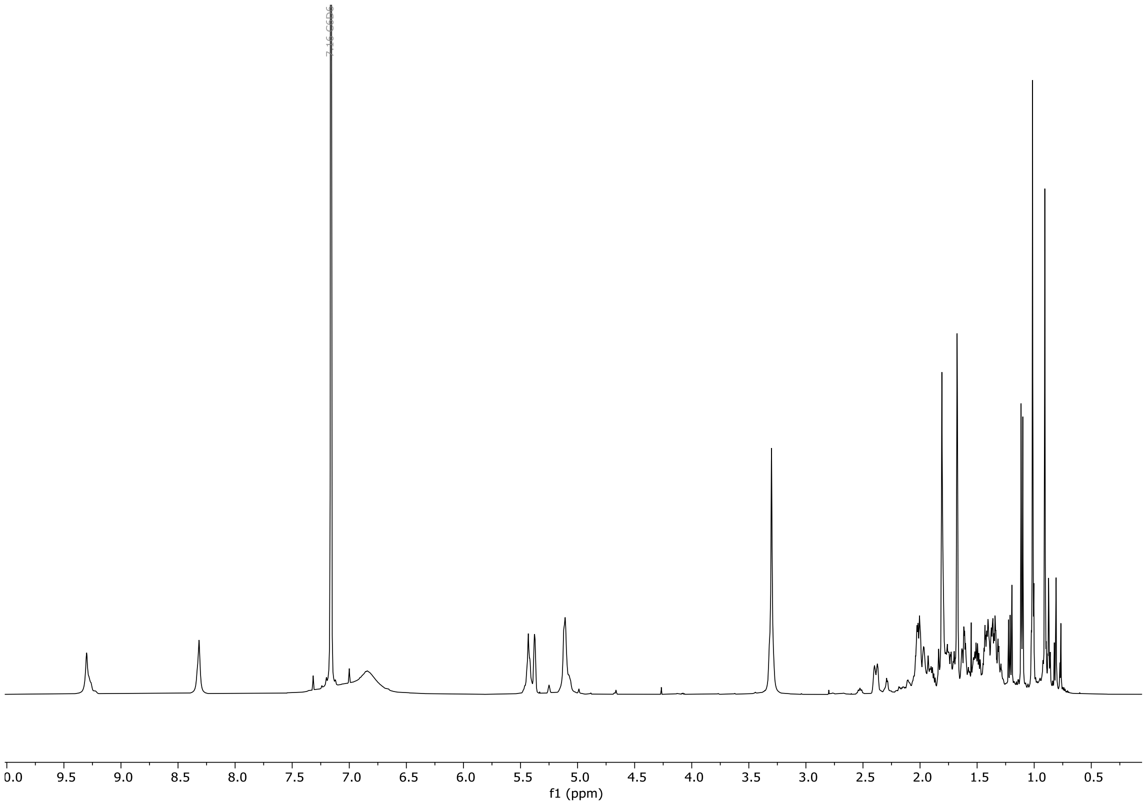

Figure S17. ^{1}H NMR spectrum (500 MHz, C_6D_6) of 10-epiagelasine B (**2**).

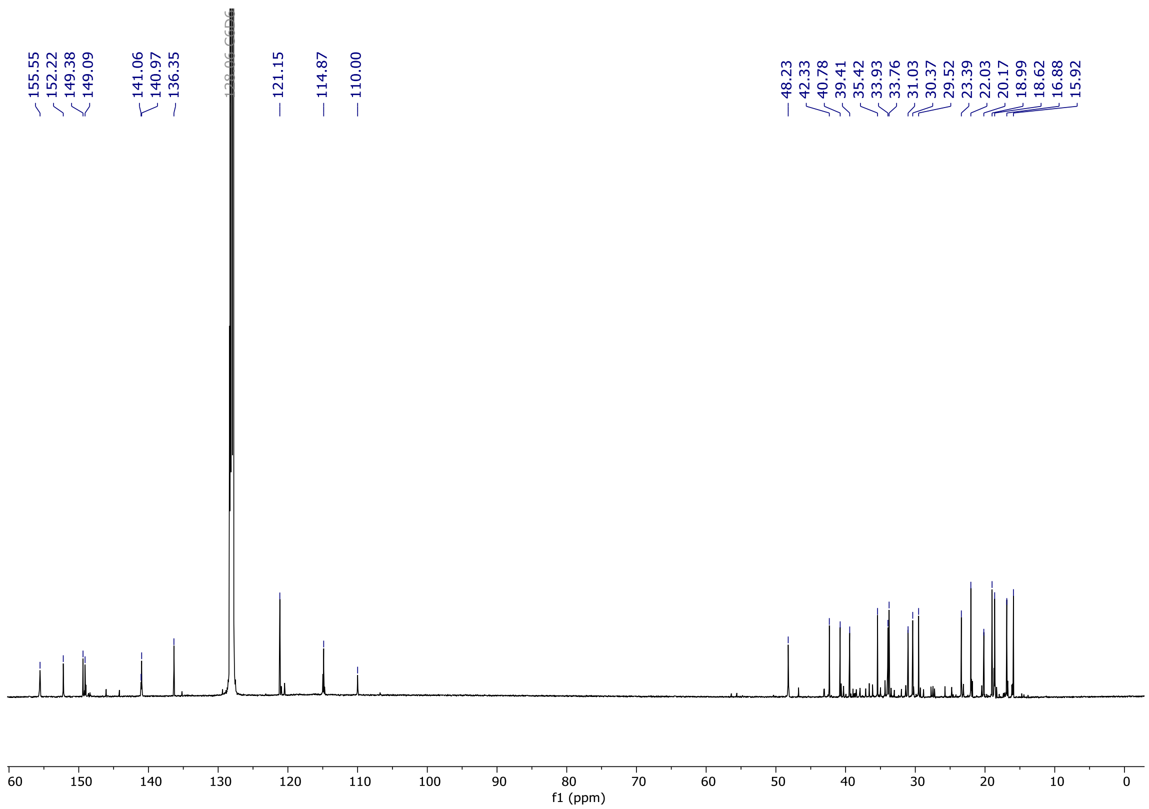

Figure S18. ^{13}C NMR spectrum (125 MHz, C_6D_6) of 10-epiagelasine B (**2**).

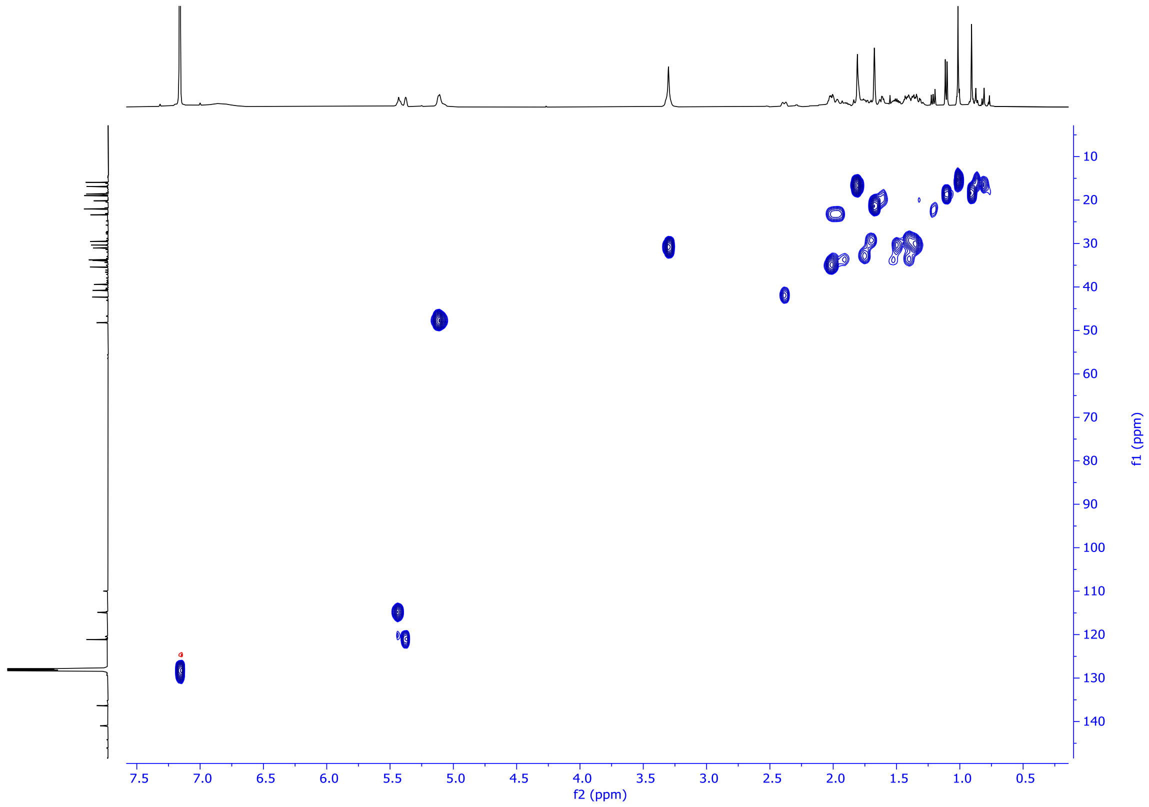

Figure S19. HSQC NMR spectrum (500 MHz, C_6D_6) of 10-epiagelasine B (**2**).

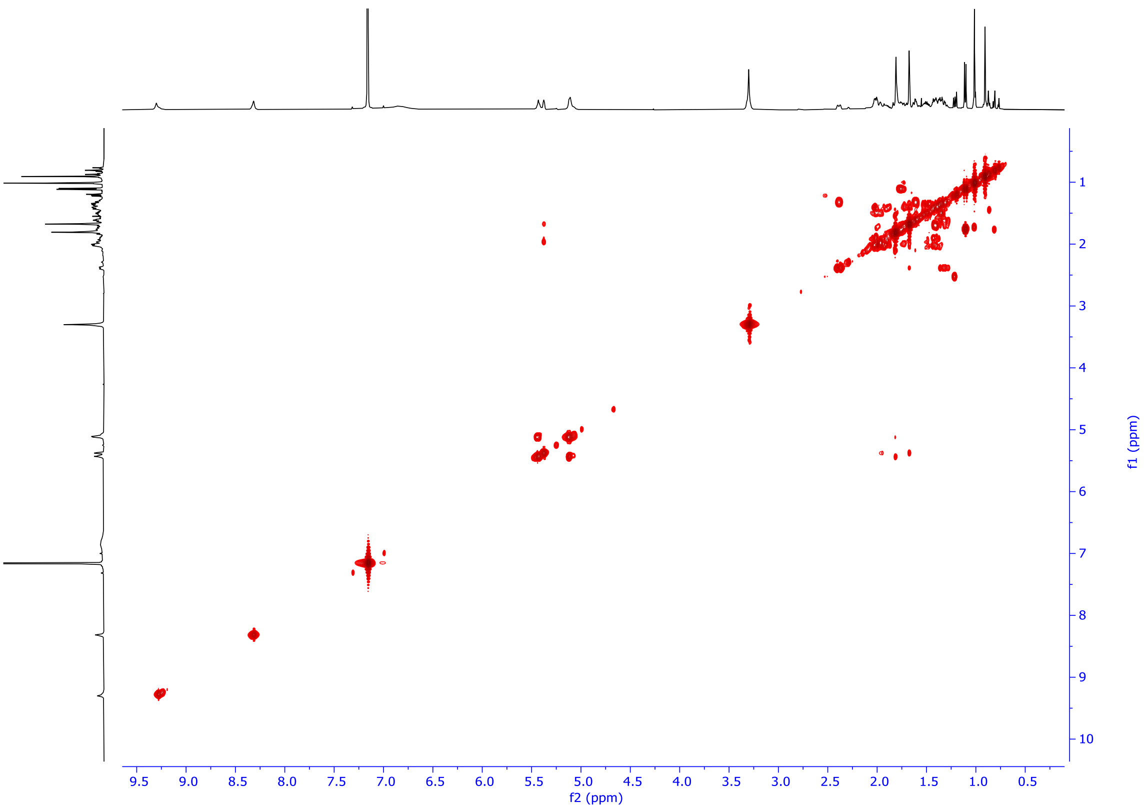

Figure S20. ^{1}H-^{1}H COSY NMR spectrum (500 MHz, C_6D_6) of 10-epiagelasine B (**2**).

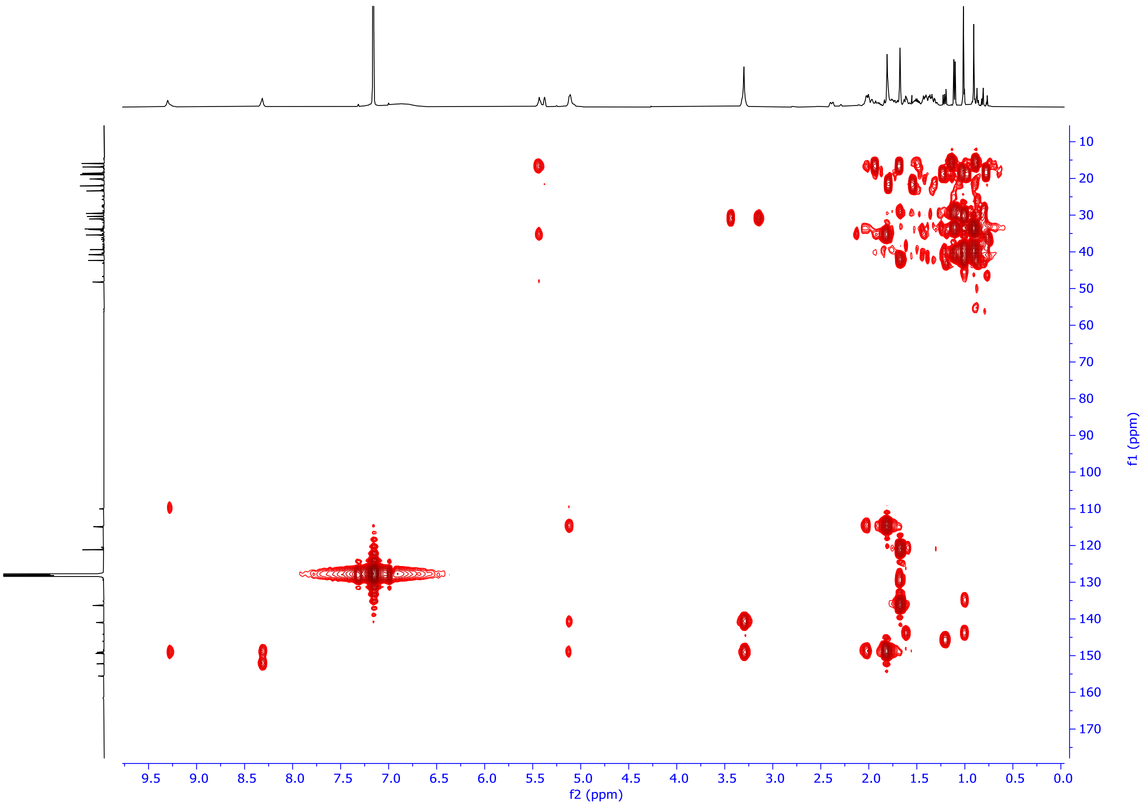

Figure S21. HMBC NMR spectrum (500 MHz, C_6D_6) of 10-epiagelasine B (**2**).

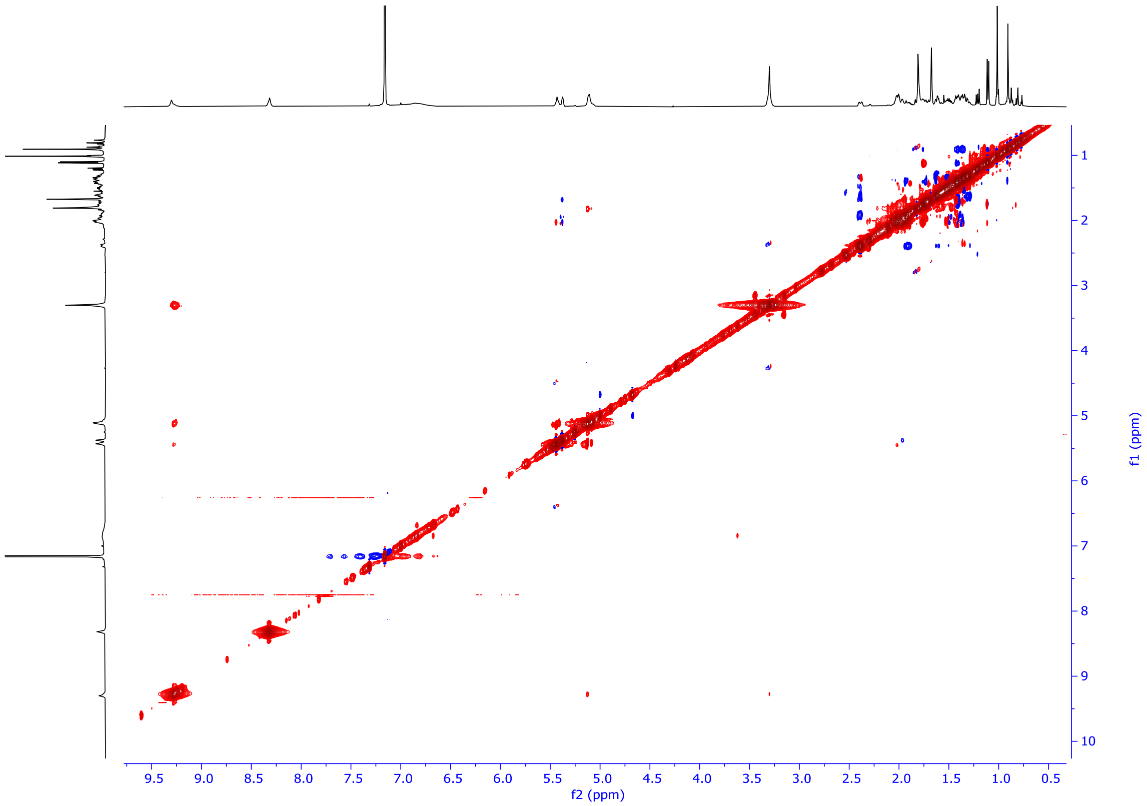

Figure S22. NOESY NMR spectrum (500 MHz, C_6D_6) of 10-epiagelasine B (**2**).

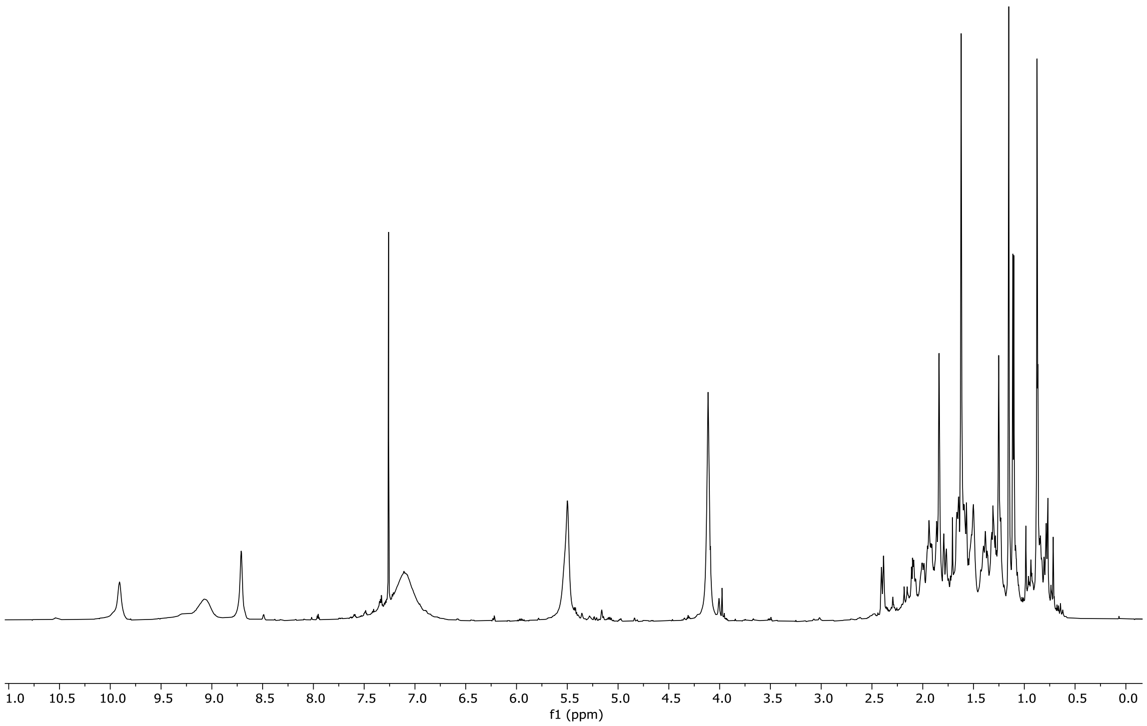

Figure S23. ^{1}H NMR spectrum (800 MHz, $CDCl_3$, after 24 h) of 10-epiagelasine B (**2**).

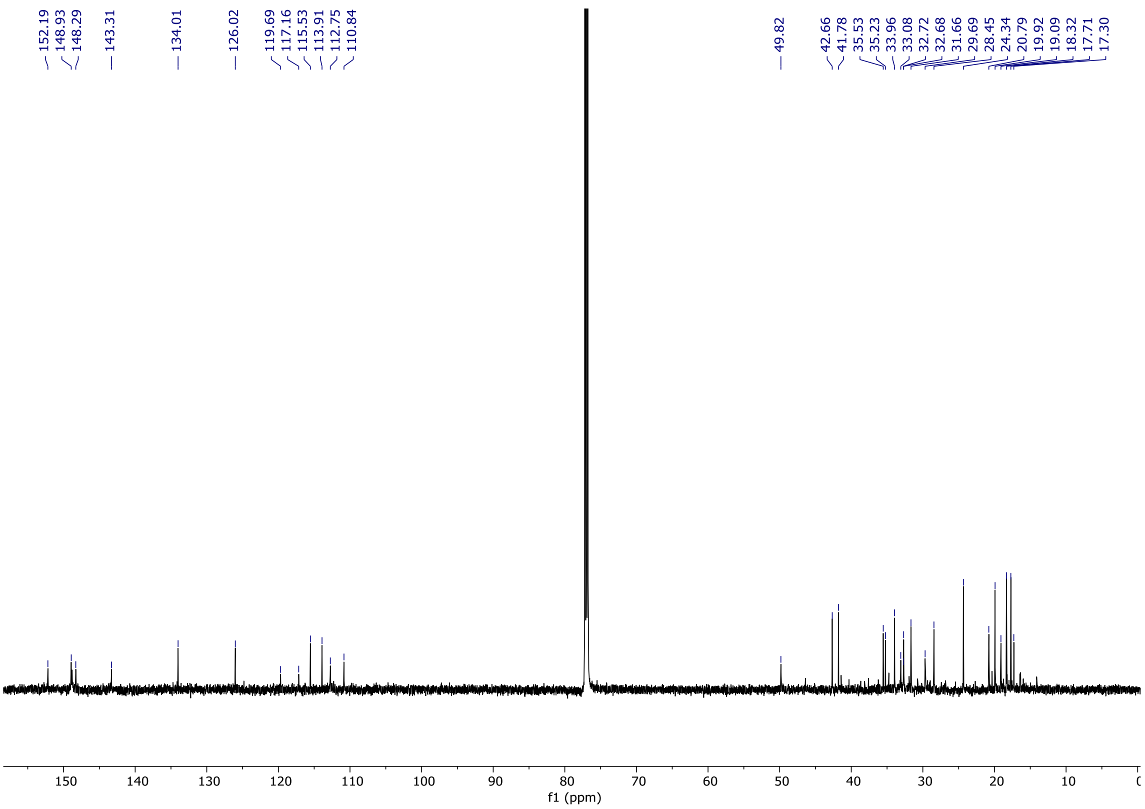

Figure S24.^{13}C NMR spectrum (200 MHz, $CDCl_3$, after 24 h) of 10-epiagelasine B (**2**).

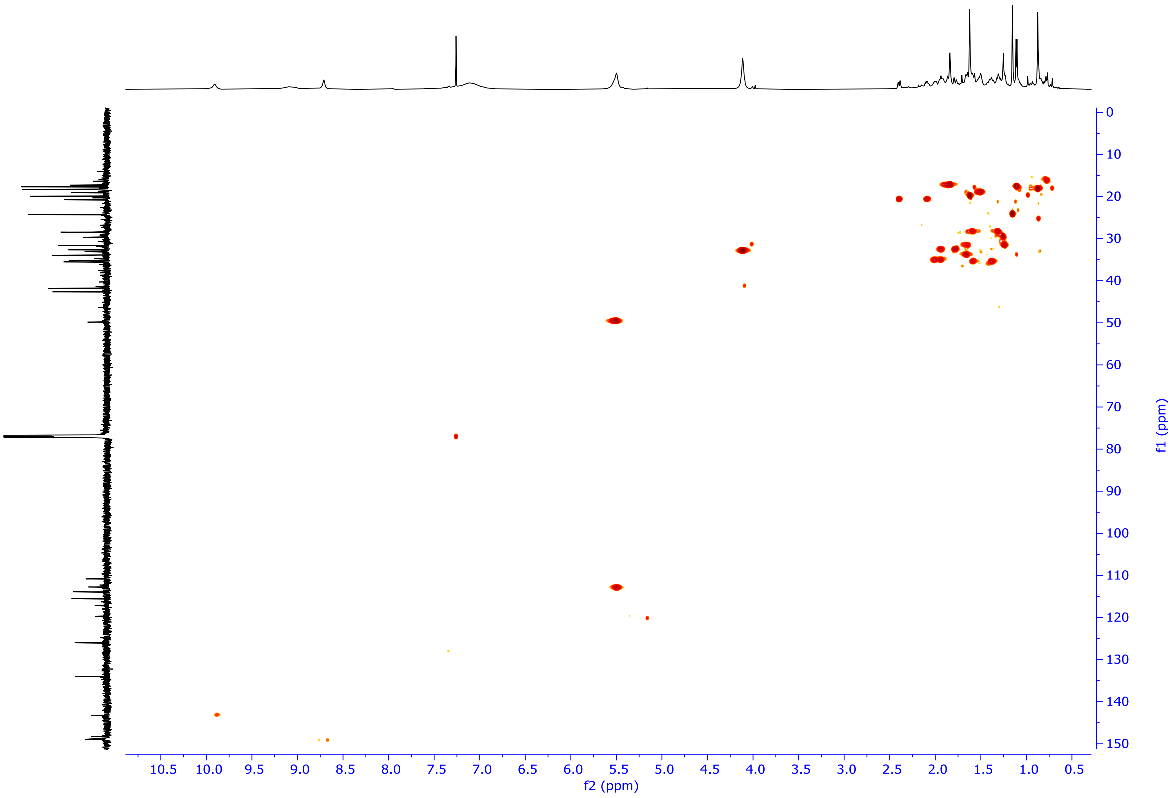

Figure S25. HSQC spectrum (800 MHz, $CDCl_3$, after 24 h) of 10-epiagelasine B (**2**). NUS parameters: 20%/384/38.

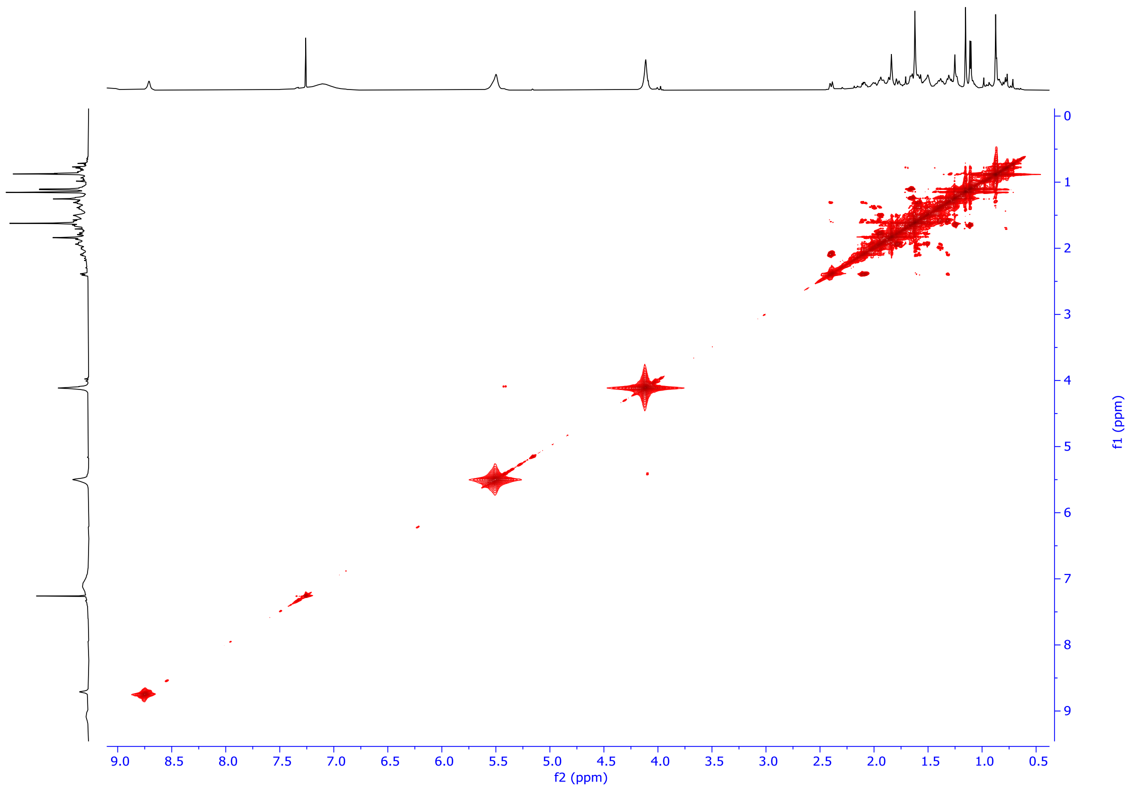

Figure S26. ^{1}H-^{1}H COSY spectrum (800 MHz, $CDCl_3$, after 24 h) of 10-epiagelasine B (**2**). NUS parameters: 50%/384/192.

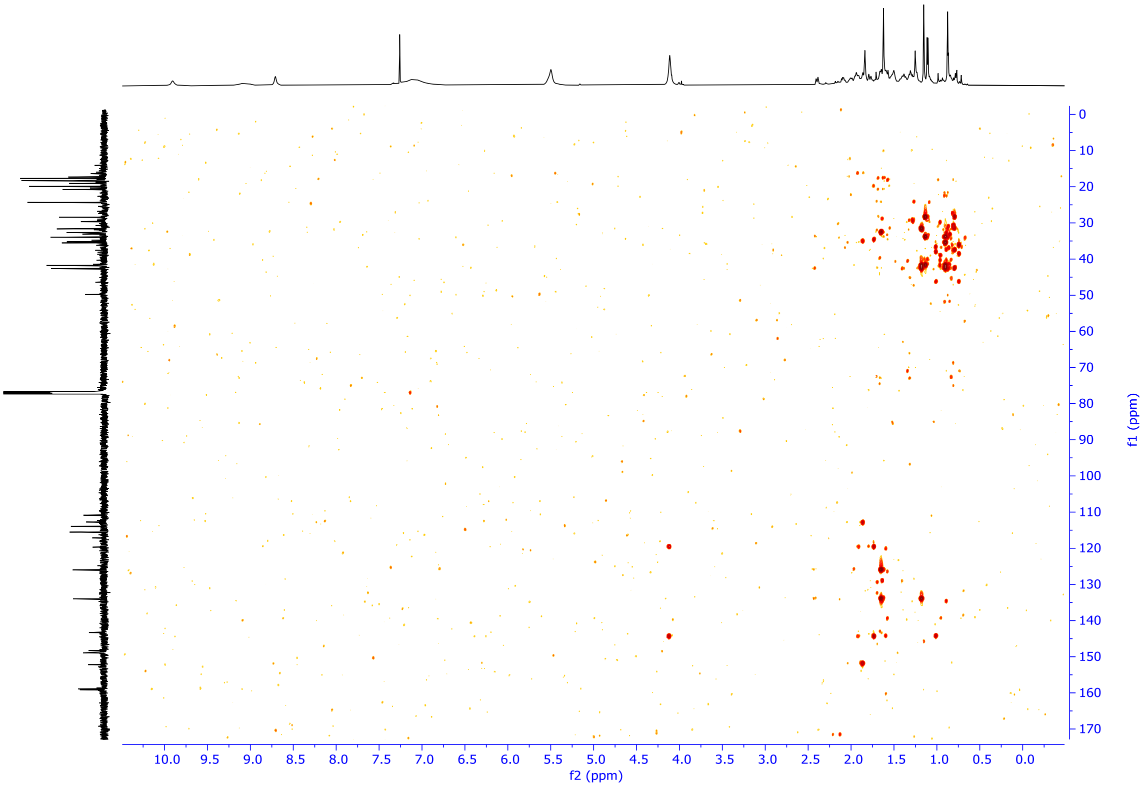

Figure S27. HMBC spectrum (800 MHz, $CDCl_3$, after 24 h) of 10-epiagelasine B (**2**). NUS parameters: 25%/512/64.

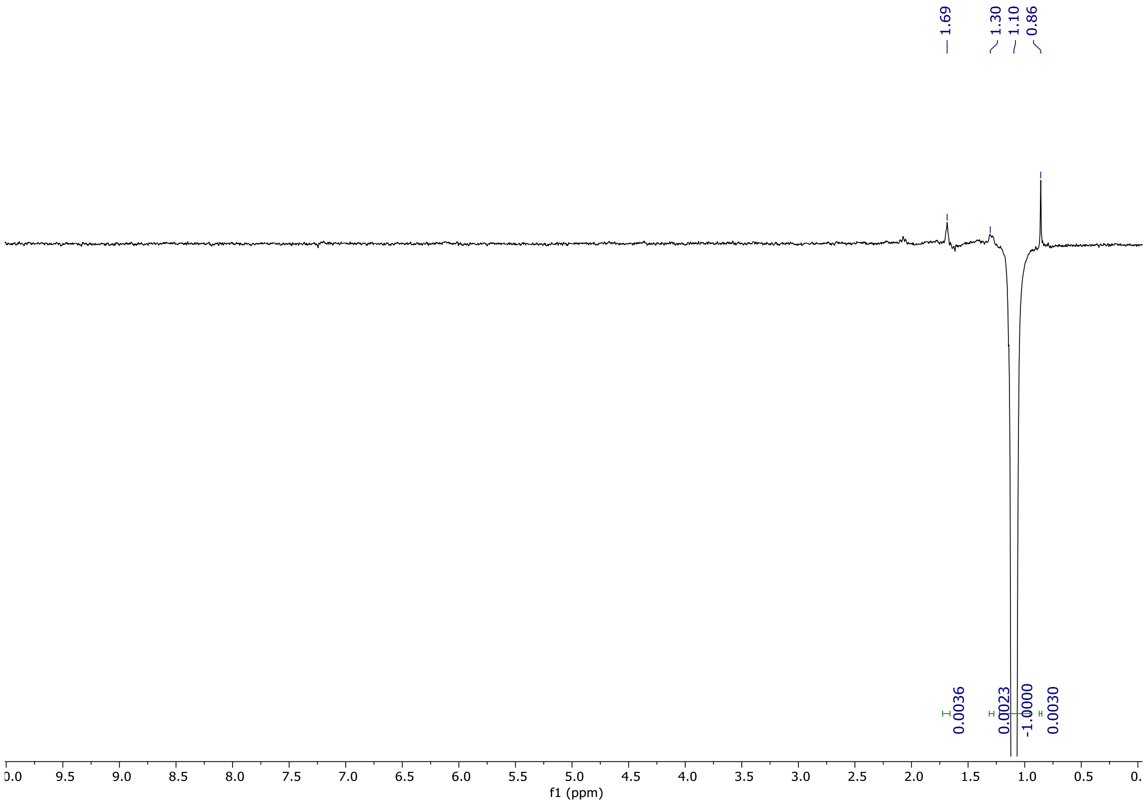

Figure S28. 1D-NOESY spectrum irradiating on CH_3-17 (δ_H 1.10) of 10-epiagelasine B (**2**) (800 MHz, $CDCl_3$, after 24 h).

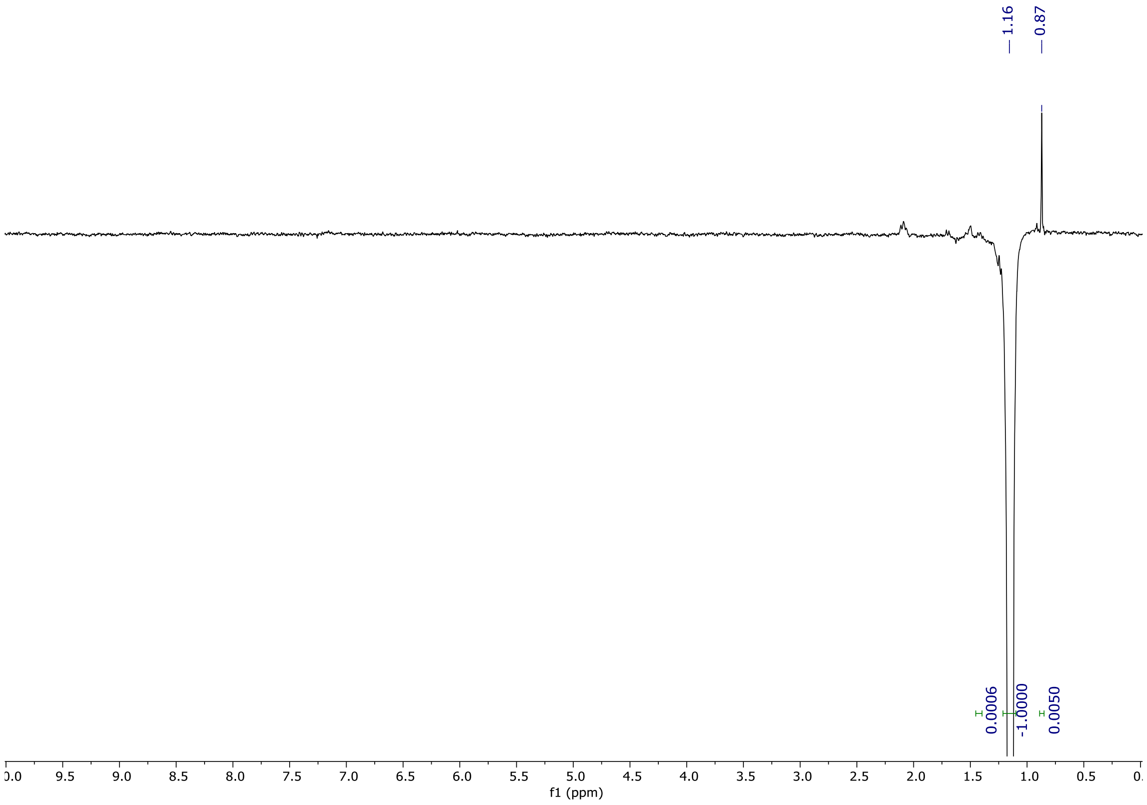

Figure S29. 1D-NOESY spectrum irradiating on CH_3-19 (δ_H 1.15) of 10-epiagelasine B (**2**) (800 MHz, $CDCl_3$, after 24 h).

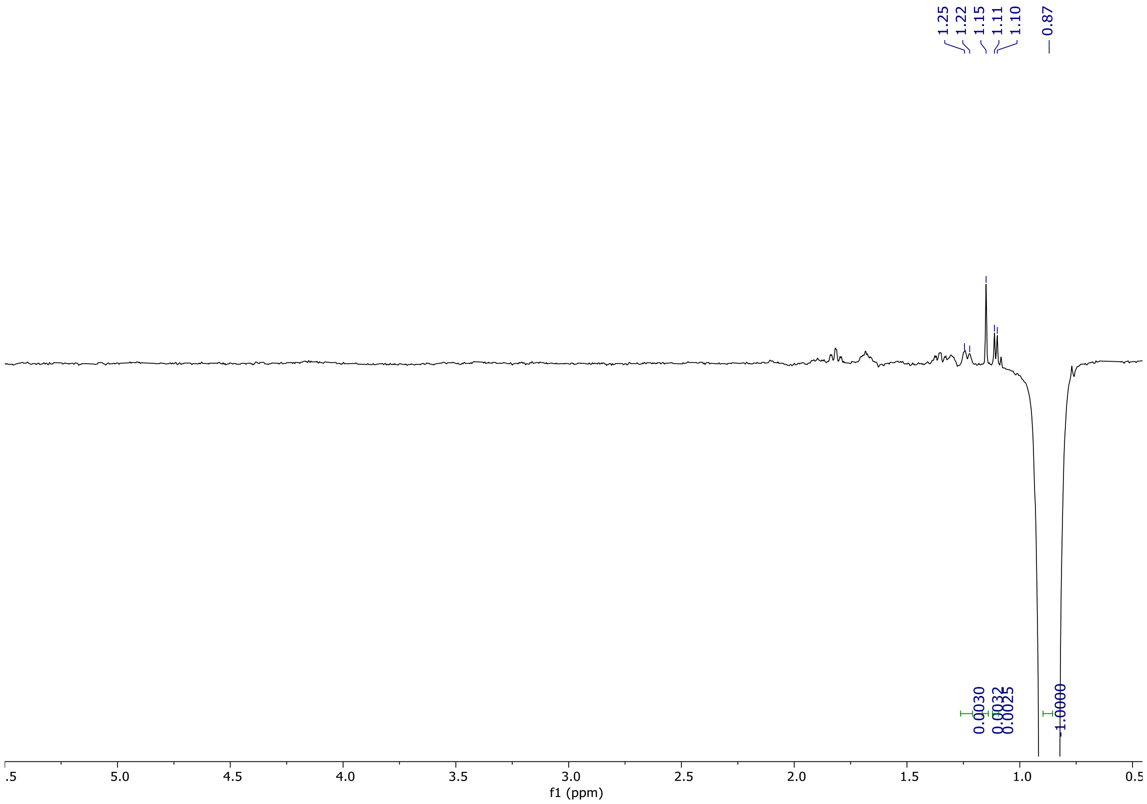

Figure S30. 1D-NOESY spectrum irradiating on CH_3-20 (δ_H 0.87) of 10-epiagelasine B (**2**) (800 MHz, $CDCl_3$, after 24 h).

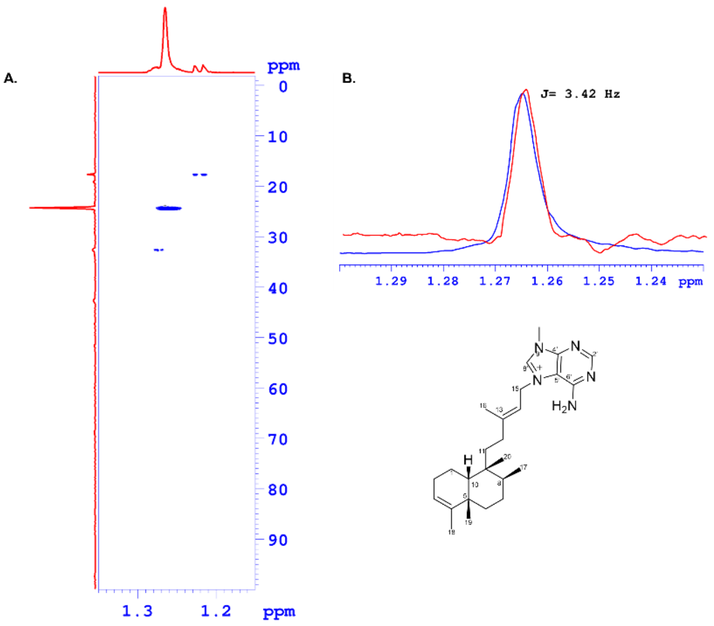

Figure S31. 2D IPAP-HSQMBC spectrum (optimized to 6 Hz) after selective inversion of H-10 proton of 10-epi-agelasine B (A). Slice from C-19 to H-10 (B). Measurement of ${}^{2,3}J_{CH}$ values were performed by analysis of IPAP multiplet patterns. NUS parameters: 12%/512/30.

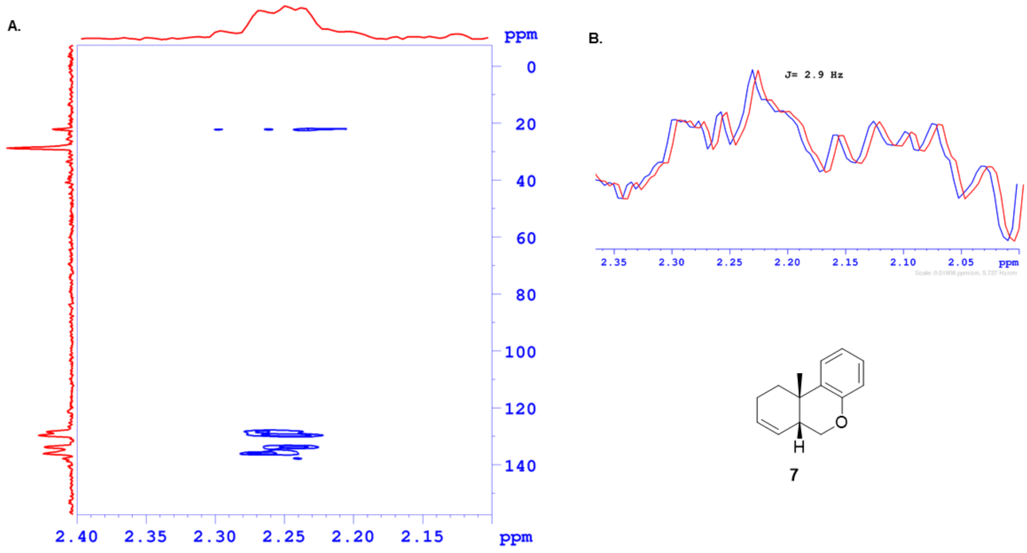

Figure S32. 2D IPAP-HSQMBC spectrum (optimized to 6 Hz) after selective inversion of H-5 proton of **7**. Slice from C-7 to H-5 (B). Measurement of ${}^{2,3}J_{CH}$ values were performed by analysis of IPAP multiplet patterns. NUS parameters: 12%/512/30.

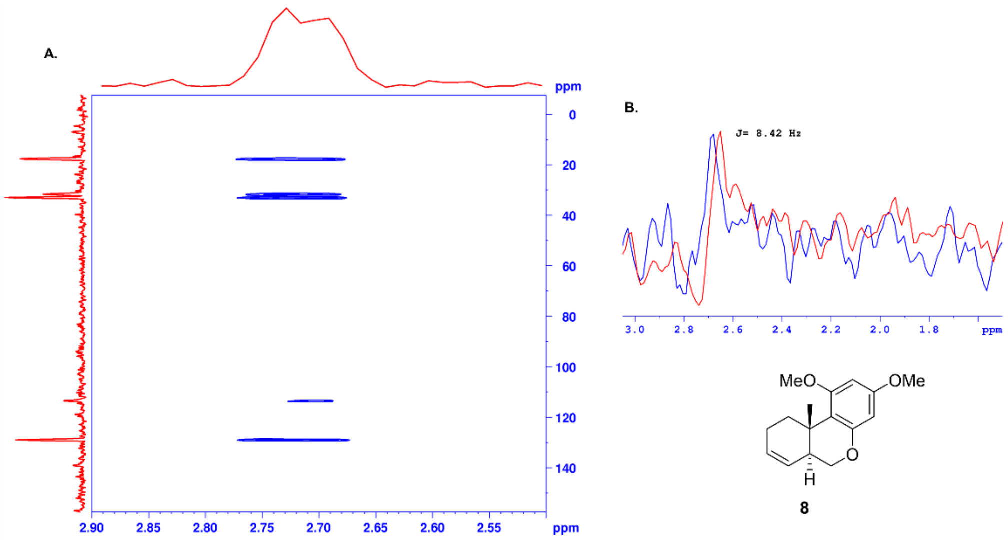

Figure S33. 2D IPAP-HSQMBC spectrum (optimized to 6 Hz) after selective inversion of H-5 proton of **8**. Slice from C-7 to H-5 (B). Measurement of $^{2,3}J_{CH}$ values were performed by analysis of IPAP multiplet patterns. NUS parameters: 12%/512/30.

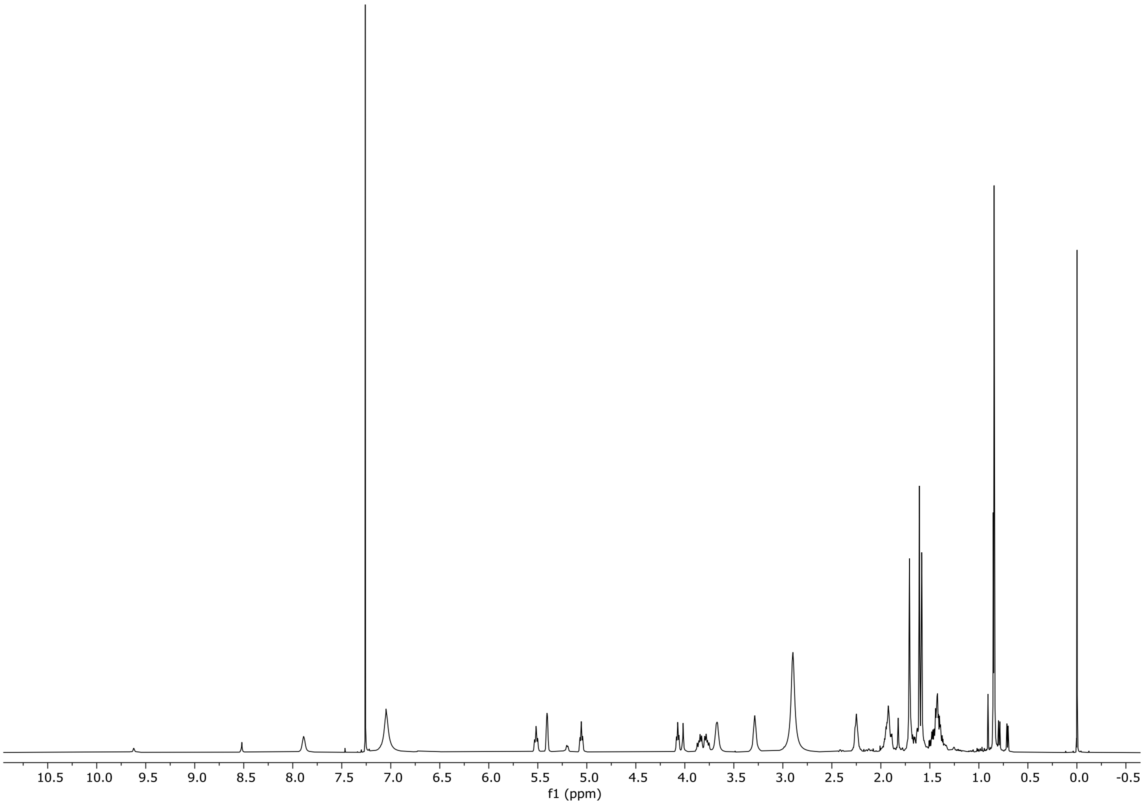

Figure S34.^{1}H NMR spectrum (500 MHz, $CDCl_3$) of 12-hydroxyagelasidine C (**3**).

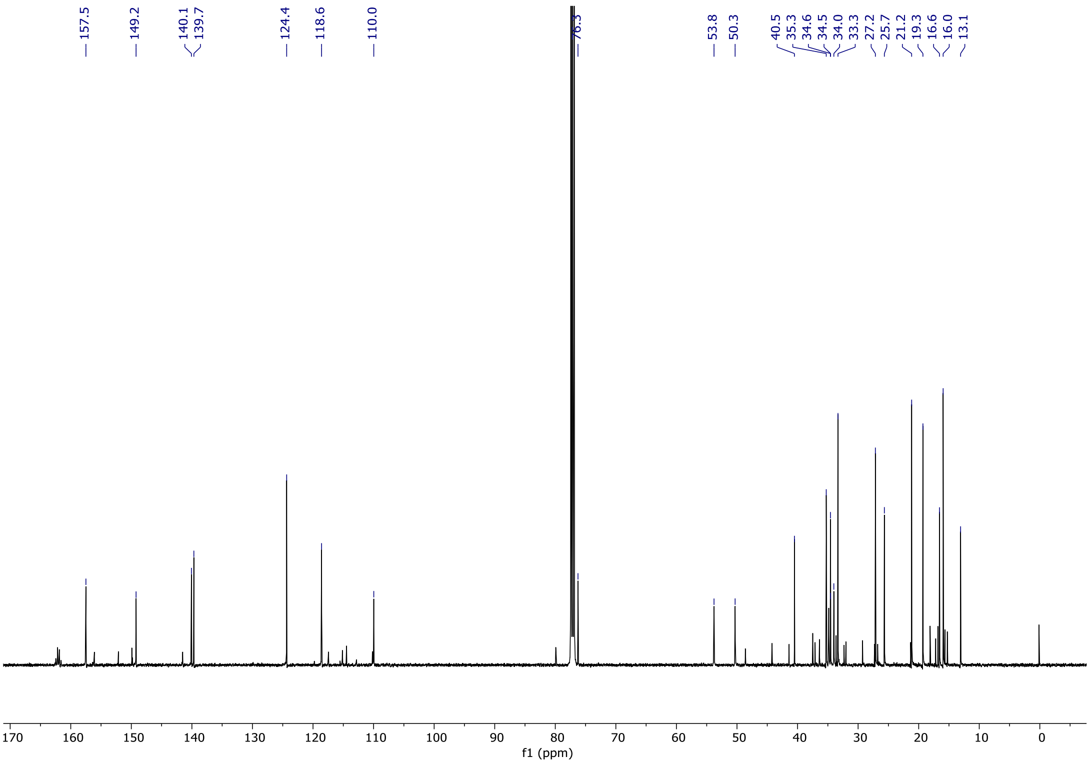

Figure S35.^{13}C NMR spectrum (125 MHz, $CDCl_3$) of 12-hydroxyagelasidine C (**3**).

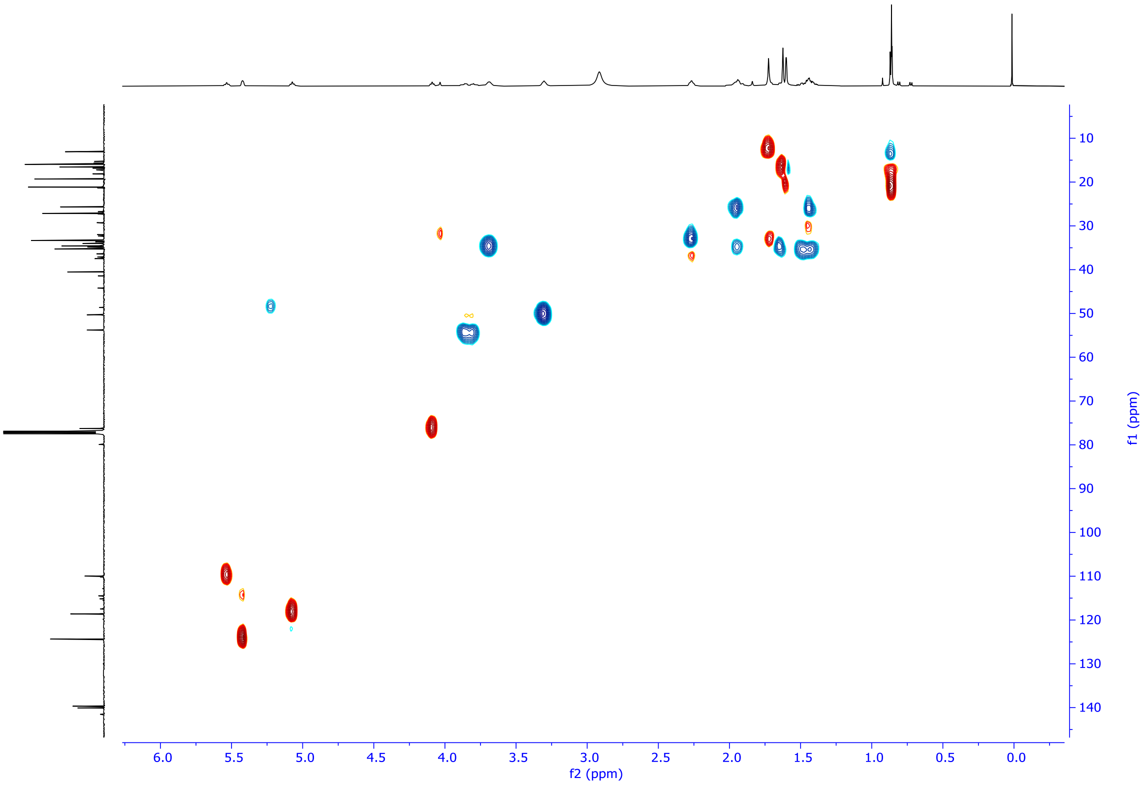

Figure S36. HSQC spectrum (500 MHz, $CDCl_3$) of 12-hydroxyagelasidine C (**3**).

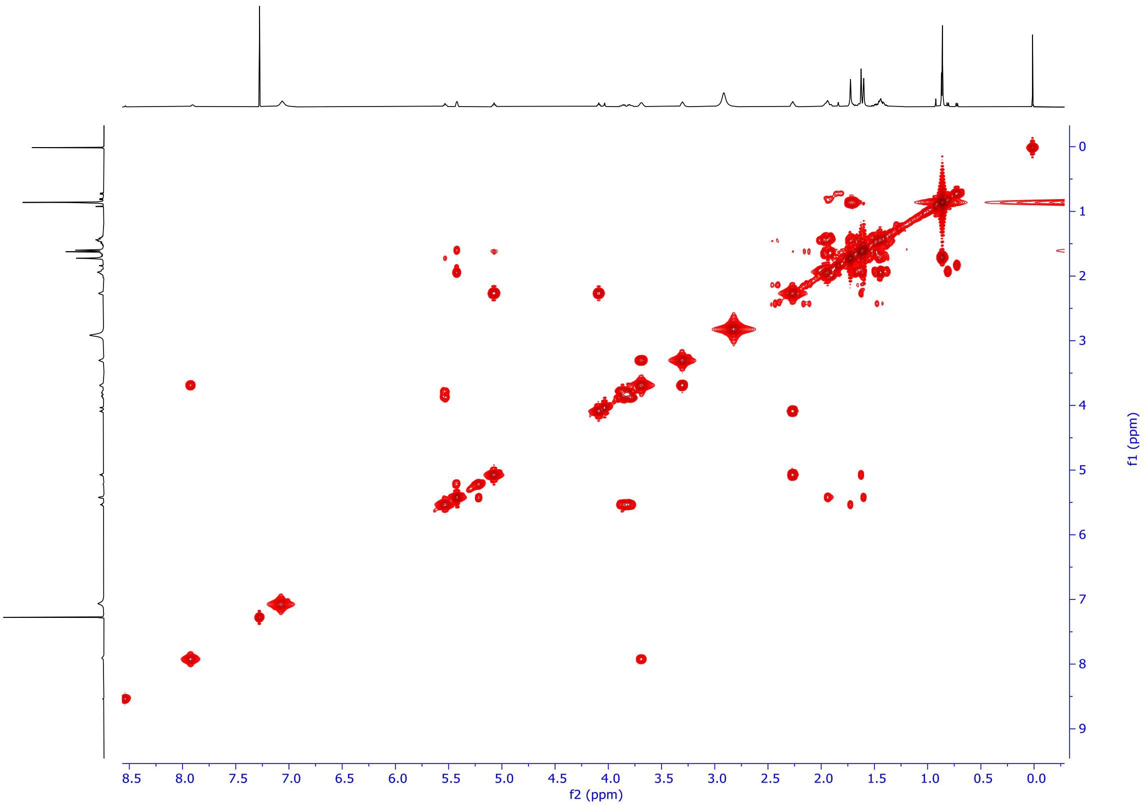

Figure S37. ^1H-^1H COSY spectrum (500 MHz, $CDCl_3$) of 12-hydroxyagelasidine C (**3**).

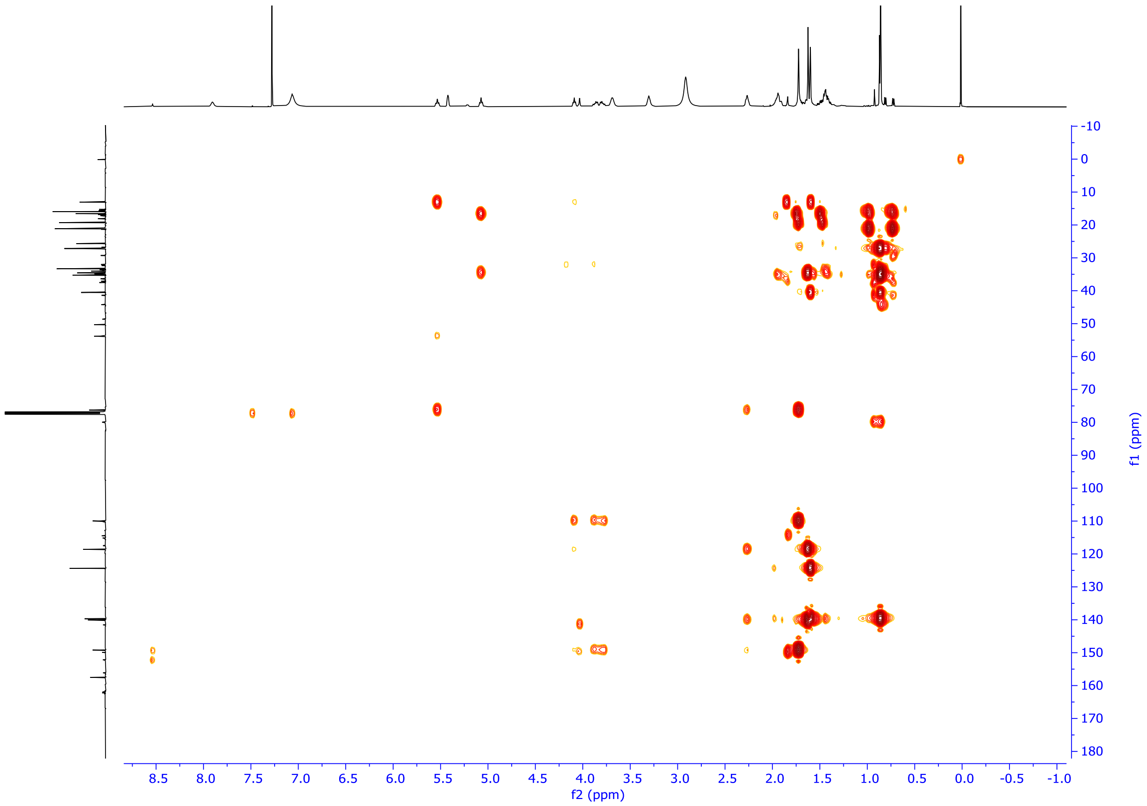

Figure S38. HMBC spectrum (500 MHz, $CDCl_3$) of 12-hydroxyagelasidine C (**3**).

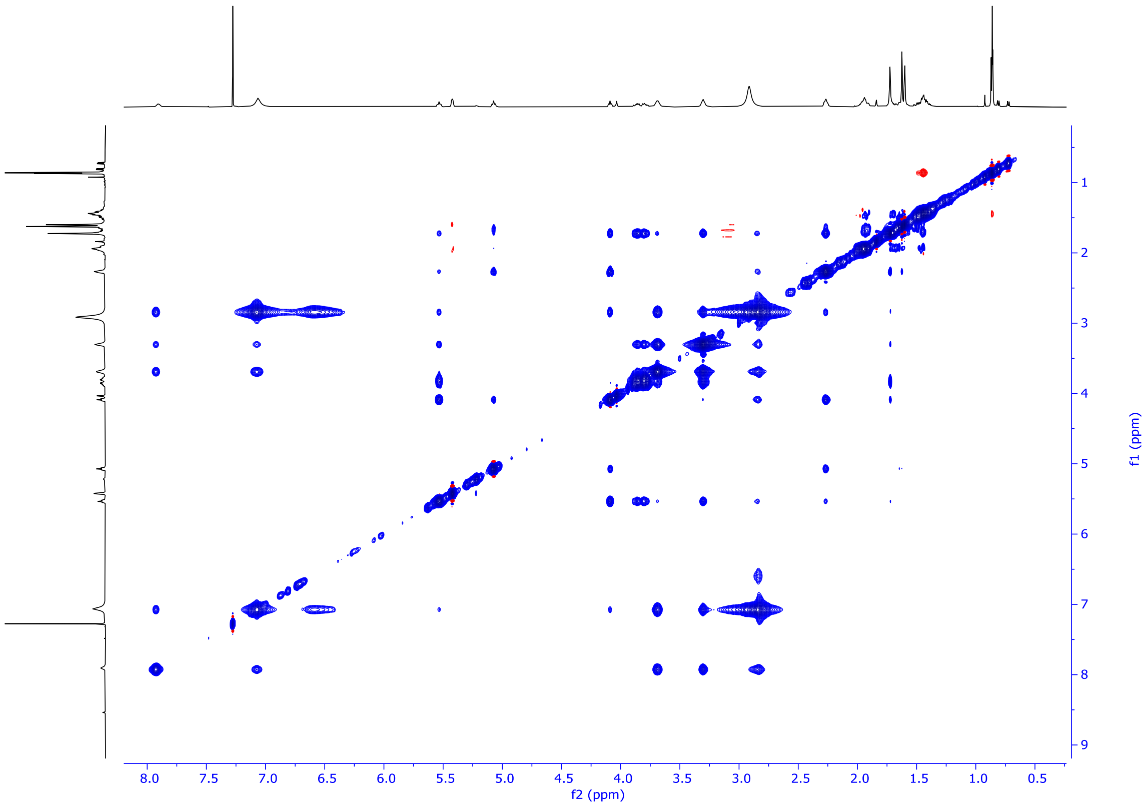

Figure S39. NOESY spectrum (500 MHz, $CDCl_3$) of 12-hydroxyagelasidine C (**3**).

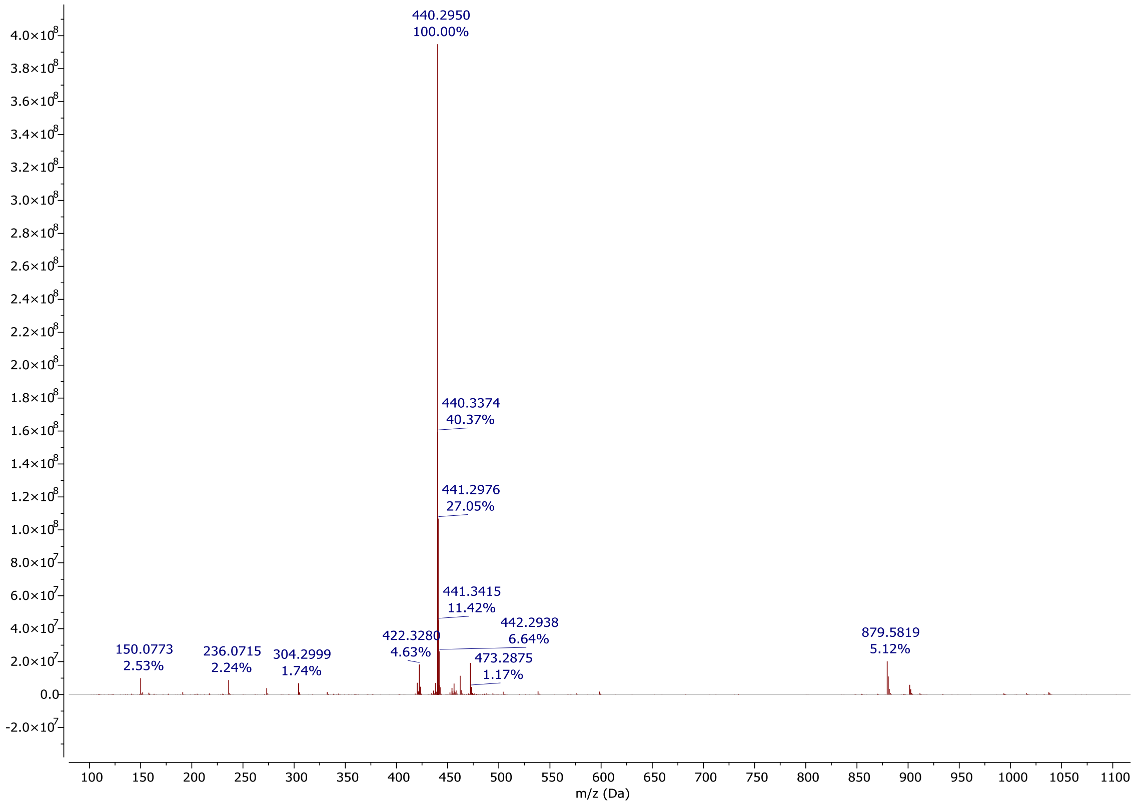

Figure S40. (+)-HR-ESIMS of 12-hydroxyagelasidine C (**3**).
